# Increased FGF8 signaling promotes chondrogenic rather than osteogenic development in the embryonic skull

**DOI:** 10.1242/dmm.031526

**Published:** 2018-06-15

**Authors:** Linnea Schmidt, Aftab Taiyab, Vida Senkus Melvin, Kenneth L. Jones, Trevor Williams

**Affiliations:** 1 Program of Reproductive Sciences and Integrated Physiology, University of Colorado Anschutz Medical Campus, Aurora, CO 80045, USA; 2 Department of Craniofacial Biology, University of Colorado Anschutz Medical Campus, Aurora, CO 80045, USA; 3 Department of Biochemistry and Molecular Genetics, University of Colorado School of Medicine, Aurora, CO 80045, USA; 4 Department of Cell and Developmental Biology, University of Colorado Anschutz Medical Campus, Aurora, CO 80045, USA; 5 Department of Pediatrics, University of Colorado Anschutz Medical Campus, Children's Hospital Colorado, Aurora, CO 80045, USA

**Keywords:** Intramembranous ossification, *Fgf8*, Cranial vault, Craniosynostosis, Osteogenesis, Chondrogenesis

## Abstract

The bones of the cranial vault are formed directly from mesenchymal cells through intramembranous ossification rather than via a cartilage intermediate. Formation and growth of the skull bones involves the interaction of multiple cell-cell signaling pathways, with fibroblast growth factors (FGFs) and their receptors exerting a prominent influence. Mutations within the FGF signaling pathway are the most frequent cause of craniosynostosis, which is a common human craniofacial developmental abnormality characterized by the premature fusion of the cranial sutures. Here, we have developed new mouse models to investigate how different levels of increased FGF signaling can affect the formation of the calvarial bones and associated sutures. Whereas moderate *Fgf8* overexpression resulted in delayed ossification followed by craniosynostosis of the coronal suture, higher *Fgf8* levels promoted a loss of ossification and favored cartilage over bone formation across the skull. By contrast, endochondral bones were still able to form and ossify in the presence of increased levels of *Fgf8*, although the growth and mineralization of these bones were affected to varying extents. Expression analysis demonstrated that abnormal skull chondrogenesis was accompanied by changes in the genes required for Wnt signaling. Moreover, further analysis indicated that the pathology was associated with decreased Wnt signaling, as the reduction in ossification could be partially rescued by halving *Axin2* gene dosage. Taken together, these findings indicate that mesenchymal cells of the skull are not fated to form bone, but can be forced into a chondrogenic fate through the manipulation of FGF8 signaling. These results have implications for evolution of the different methods of ossification as well as for therapeutic intervention in craniosynostosis.

## Abstract (precis)

**Summary:** Cranial ossification responds to *Fgf8* overexpression in a dose-dependent manner with moderate levels leading to craniosynostosis and higher levels shifting cranial vault ossification to abnormal cartilage formation.

## INTRODUCTION

Bone forms via two processes: intramembranous or endochondral ossification. The majority of the skeleton, including the long bones, vertebrae and basicranium, forms via endochondral ossification during which condensed mesenchyme cells first differentiate into chondrocytes that form cartilage tissue. This intermediate cartilaginous template is then replaced by bone, formed through osteogenesis. By contrast, most of the skull, including the jaw and cranial vault, is generated via intramembranous ossification, in which condensed mesenchyme cells directly differentiate into osteoblasts that form bone without any cartilaginous precursor (Ornitz and Marie, 2015). The anterior cranial vault, comprising the frontal bones, is derived from cranial neural crest cells (Couly et al., 1993; Jiang et al., 2002), whereas the posterior cranial vault bones are derived from either the paraxial mesoderm (parietal) or a combination of paraxial mesoderm and cranial neural crest cells (interparietal/occipital) (Jiang et al., 2002; Noden, 1992; Yoshida et al., 2008). Together, these cells derived from the cranial neural crest and paraxial mesoderm form the intramembranous bones and sutures of the skull (Evans and Noden, 2006; Jiang et al., 2002; Noden and Trainor, 2005; Opperman, 2000). Intramembranous ossification of the skull vault involves direct bone matrix deposition to form calvarial plates, which expand during development but do not fuse with other cranial bones during embryogenesis and infancy (Hall and Miyake, 2000). Instead, sutures connect the individual intramembranous bones and serve as growth centers that regulate the expansive growth of the skull (Nie et al., 2006). Because sutures are the major sites of bone growth during cranial vault development, signaling at the sutures is essential for the regulation of intramembranous ossification (Cohen, 2000; Ornitz and Marie, 2002). Several signaling pathways are implicated in proper skull ossification and growth, including the fibroblast growth factor (FGF), hedgehog (HH) and Wnt signaling (Katsianou et al., 2016) pathways. The role of FGF signaling in ossification is of particular interest, as mutations in the FGF signaling pathway, which comprises four fibroblast growth factor receptors (FGFRs) and 22 FGF ligands (Brewer et al., 2016; Pownall and Isaacs, 2010), cause a number of skeletal disorders (Nie et al., 2006; Ornitz and Marie, 2015). These disorders include those that affect cranial vault ossification, such as craniosynostosis.

Craniosynostosis is the second most common human craniofacial abnormality, occurring in 1 in 2100 to 2500 births (Boulet et al., 2008; Lajeunie et al., 1995). The disorder is characterized by the premature fusion of the metopic, sagittal, lambdoid and/or coronal sutures, which causes a distortion in skull shape that frequently requires surgical correction. Activating mutations in the FGF signaling pathway, mostly heterozygous mutations of FGFRs, account for the majority of known causes of craniosynostosis (Johnson and Wilkie, 2011). The etiology of craniosynostosis is complicated, however, by the fact that activating mutations in the same gene can cause a variety of different syndromes and/or types of craniosynostosis; for example, mutations in *FGFR2* can cause seven of the eight FGFR-related craniosynostosis disorders, including Apert, Crouzon and Pfeiffer syndromes (Moosa and Wollnik, 2016; Robin et al., 2011). This diversity, in part, reflects how each mutation affects the multiple signaling pathways that are directly regulated by FGFRs, as well as the potential for genetic interactions to affect the phenotype (Ratisoontorn et al., 2003).

Detailed studies using genetically modified mice with dominant or loss-of-function FGFR mutations have also confirmed the central role of this receptor family in bone formation, skeletal development and craniosynostosis (Su et al., 2014). With respect to the FGF ligands, FGF2, 9 and 18 have been shown to act in normal mouse cranial vault ossification (Coffin et al., 1995; Hung et al., 2016; Liu et al., 2002; Ohbayashi et al., 2002). Moreover, mouse mutations that increase the diffusion properties and effective range of FGF9 also lead to craniosynostosis (Spicer, 2009). This latter result might reflect the mechanism underlying human multiple synostoses syndrome, which is caused by rare autosomal mutations in FGF9 (Wu et al., 2009). Taken together, these studies indicate that the molecular properties of FGF ligands and receptors, along with dosage, location and timing, probably all contribute to the complexity of craniosynostosis etiology.

The specific mechanisms by which FGF signaling affects skull development and craniosynostosis remain poorly understood, however. Notably, mice heterozygous or homozygous for activating mutations in FGFRs have different craniofacial phenotypes, with homozygous gain-of-function mutations often phenocopying aspects of the loss-of-function phenotype (Mai et al., 2010; Snyder-Warwick et al., 2010). Moreover, activating mutations in FGFRs can lead to both ossification of the sutures as well as thinner calvaria (Tholpady et al., 2004). To further investigate the mechanisms underlying these somewhat contradictory developmental abnormalities, we have generated mouse models that can be induced to overexpress different levels of *Fgf8b* in the ectoderm of the developing skull. The choice of the FGF8 ligand was based both on its ability to influence development and patterning in multiple contexts as well as its homology to FGF18, which has an important role in chondrogenesis and osteogenesis (Hung et al., 2016; Liu et al., 2007, 2002; Ohbayashi et al., 2002). Further, we employed the *Fgf8b* isoform because this is more potent than *Fgf8a* in many developmental processes, a finding which correlates with the higher affinity of FGF8B for FGF receptors (Meyers et al., 1998; Olsen et al., 2006; Zeller et al., 2009). By comparing skeletal formation in these new mouse models we demonstrate that the dosage of *Fgf8b* expression differentially affects development of the calvaria and the associated sutures. Low *Fgf8b* expression mimics the craniosynostosis seen with particular FGFR2-activating mutations. By contrast, higher *Fgf8b* expression severely disrupts the process of intramembranous bone formation. Overall, these results provide insight into the mechanisms and potential treatment of craniosynostosis and further our understanding of the function of FGFs in skeletal development and ossification.

## RESULTS

### Generation of new alleles for differential expression of *Fgf8*

**Fig. 1.**
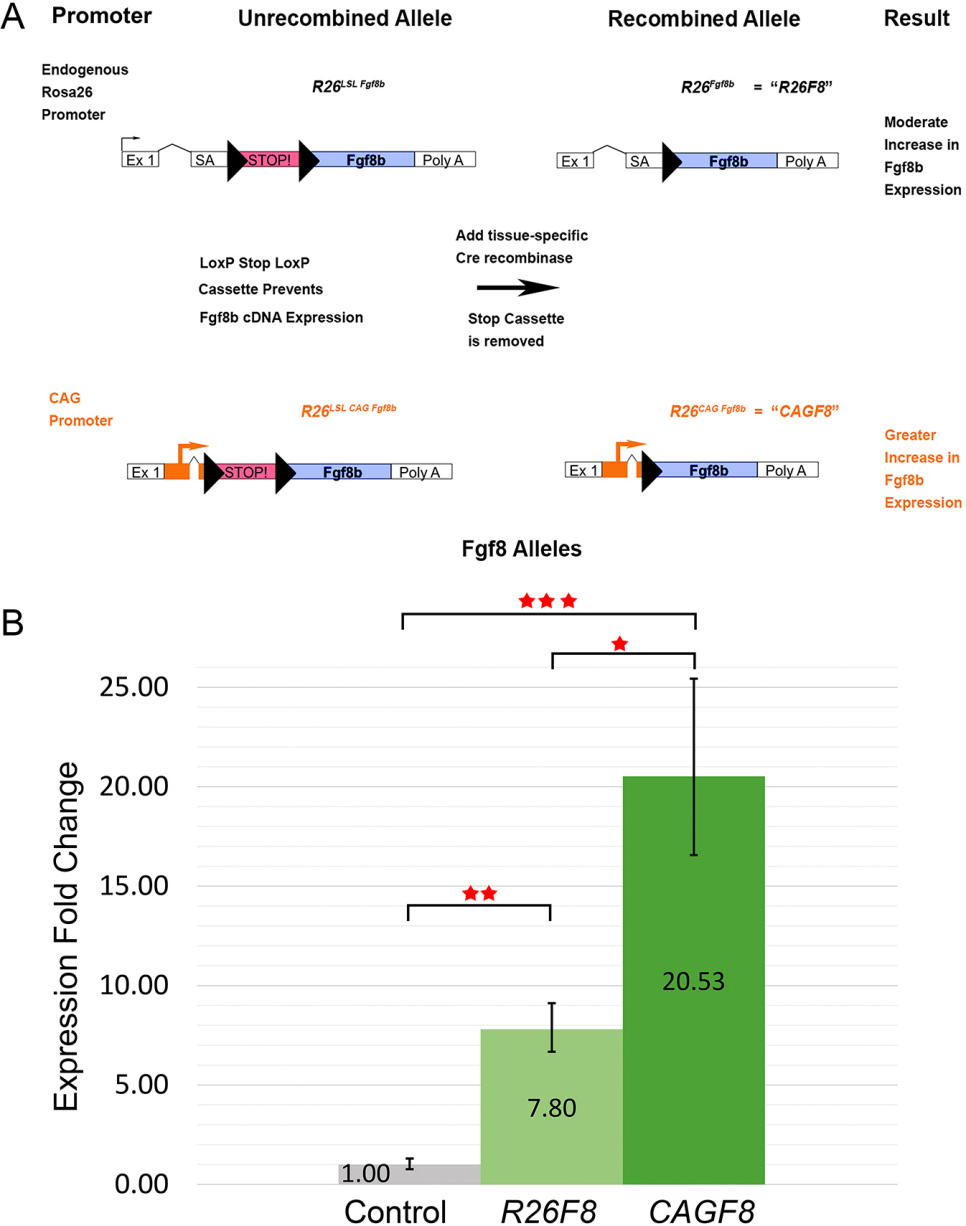
**Conditional alleles modulate *Fgf8b* expression levels.** (A) Cartoon diagram of the two *Fgf8b* conditional alleles placed within the mouse *Gt(ROSA)26Sor* locus before and after Cre-mediated recombination with exons (Ex), splice acceptors (SA) and *LoxP* sites (black triangles) illustrated. The abbreviations *R26F8* (top) and *CAGF8* (bottom) are used to refer to these recombined alleles throughout the text of the article. (B) Relative *Fgf8b* expression levels in E18.5 dorsal head skin measured by qPCR, normalized to *Actb* levels, following *Msx2-Cre*-mediated recombination. Control indicates data from littermates lacking *Msx2-Cre* transgene. Bars show average fold change for each group compared with the control, with error bars indicating standard error. All three groups are statistically significant from each other (Anova: *P*<0.001; *t*-test: controls versus *R26F8*, **P=0.0013; controls versus *CAGF8*, ***P=0.0002; *R26F8* versus *CAGF8*, *P=0.022). Controls, *n*=6; *R26F8* and *CAGF8*, *n*=3.

Two new alleles were generated to manipulate the expression of *Fgf8* transcripts *in vivo*. The first allele incorporates the *Fgf8b* cDNA into the *Gt(ROSA)26Sor* locus under the control of a Lox-Stop-Lox cassette (Fig. 1A, Fig. S1A). Breeding mice with this *R26^LSL Fgf8b^* allele to mice expressing a Cre recombinase transgene allows the deletion of the Stop cassette to generate a new allele expressing the *Fgf8b* cDNA (*R26^Fgf8b^*), hereafter termed *R26F8*. To ascertain the effects of higher levels of *Fgf8* overexpression, we generated an additional construct: *R26^LSL CAG Fgf8b^* (Fig. 1A, Fig. S1B). Following Cre-mediated recombination this would generate the *R26^CAG Fgf8b^* allele, hereafter termed *CAGF8*. We initially focused on how elevated FGF ligand expression in the overlying ectoderm could influence the development of the underlying mesenchyme at different time points and in different locations by testing a number of Cre recombinase transgenes that target the embryonic ectoderm. In the current study, we focused on formation and patterning of the craniofacial skeleton and determined that *Msx2-Cre* had a spatiotemporal pattern of expression that was the most suitable to study calvarial development (Sun et al., 2000; Choe et al., 2012) (Fig. S2). Specifically, when used in combination with *R26 LacZ* reporter mice, *Msx2-Cre* activity was detected in the head ectoderm beginning around embryonic day (E) 10.5, but was not detected in the underlying mesenchyme derivatives of the skull including the bones and sutures (Fig. S2). We next used quantitative real-time PCR (qPCR) to examine how *Fgf8b* mRNA levels were altered when *Msx2-Cre* was employed with either of the new *Fgf8* alleles (Fig. 1B). At E18.5, in the presence of *Msx2-Cre*, *Fgf8b* expression was increased ∼8 times in *R26F8* mutants (Fig. 1B, *P*=0.0013) and ∼21 times in *CAGF8* mutants compared with controls (Fig. 1B, *P*=0.0002). Therefore, both the *R26F8* and *CAGF8* alleles increase *Fgf8b* mRNA expression when activated by a Cre recombinase transgene, with the latter generating ∼2.5-fold more transcript than the former (Fig. 1B, *P*=0.022).

### Moderately increased levels of *Fgf8* transcripts cause craniosynostosis

**Fig. 2.**
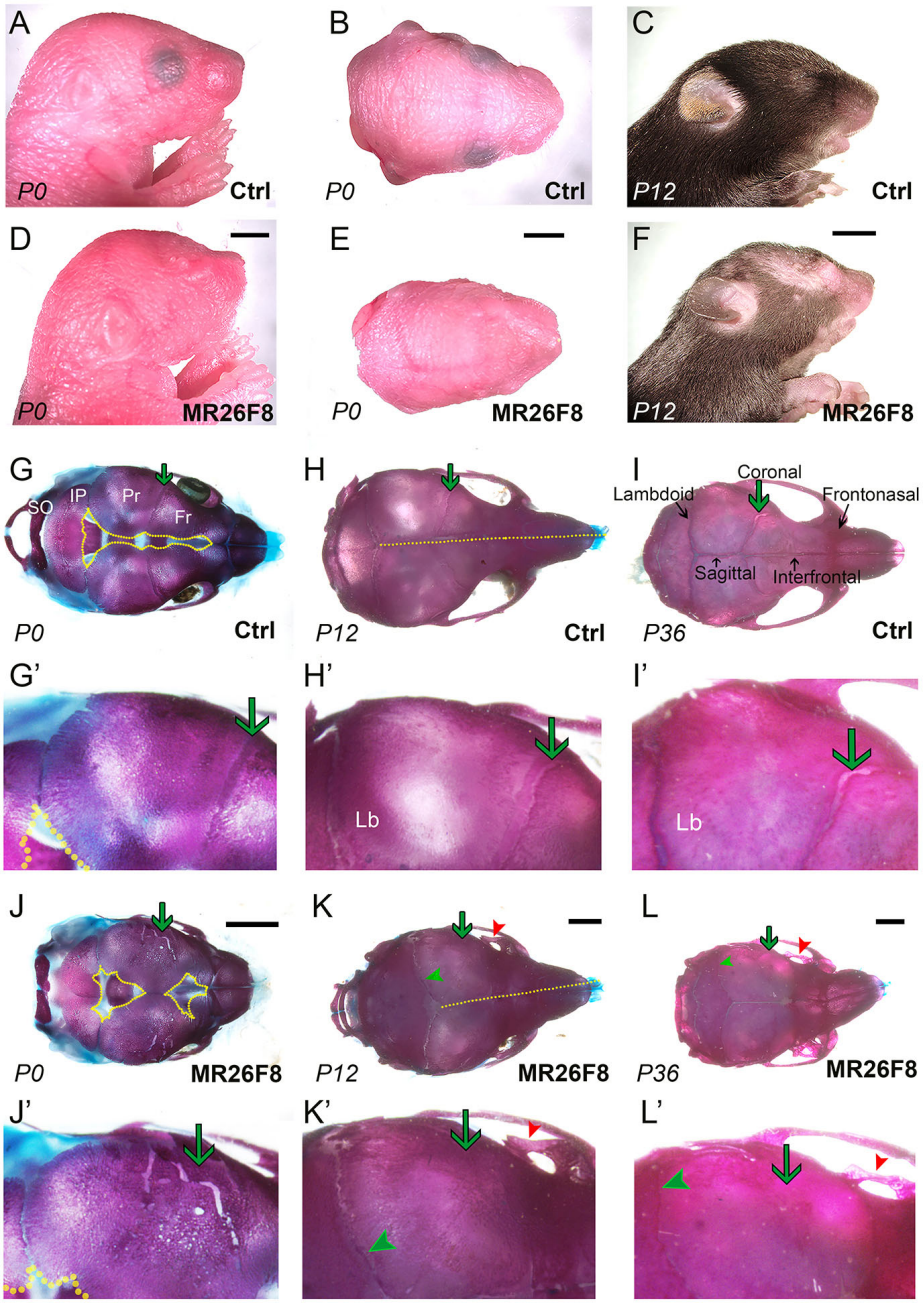
***MR26F8* mice exhibit cranial defects including coronal craniosynostosis.** (A-F) Gross images of the control (A-C) and *MR26F8* (D-F) heads. Neonate pups (P0) are shown in both a lateral (A,D) and dorsal (B,E) view. P12 heads are shown laterally (C,F). (G-L) Dorsal views of bone and cartilage staining of control (G-I) and mutant (J-L) skulls at P0 (G,J), P12 (H,K) and P36 (I,L). G′-L′ show magnification of the coronal and lambdoid sutures of each respective skull. The expected location of the coronal suture is shown by a green arrow. This suture is present in controls, abnormal in *MR26F8* mice at P0 and absent at P12 and P36. The narrower region of the *MR26F8* lambdoid suture at P12 and P36 (green arrowheads) and ectopic bone (red arrowheads) are shown. The yellow dotted lines in G and J outline the edges of ossification. The yellow dotted lines in K and L align with the sagittal and interfrontal sutures. Fr, frontal bone; Lb, lambdoid suture; IP, interparietal bone; Pr, parietal bone; SO, supraoccipital bone. Scale bars: (D,E,J-L) 1 mm; (F) 5 mm. Aged-matched controls and mutants are at the same scale. A-F, *n*=10; G-L, *n*=5.

We next studied development in mice with a moderate increase in *Fgf8b* expression. These *Msx2-Cre;R26^Fgf8b^* mice, hereafter termed *MR26F8*, were born at normal size and in normal Mendelian ratios, but were easily identifiable from mid-gestation onwards by their gross morphology. The *MR26F8* mice had both striking craniofacial defects (Fig. 2A-F, Fig. S3A,B, Fig. S4) as well as limb defects including sirenomelia and post-axial polydactyly (Fig. S3C-L), the latter phenotypes consistent with the expression of the *Msx2-Cre* transgene in the limb bud ectoderm (Sun et al., 2000). The facial abnormalities included shortened snout, abnormal skin with raised rounded protrusions that extended to cover the eye (Fig. 2D-F, Fig. S3B) and patchy or absent hair on top of the head, as well as on the limbs and underbelly (Fig. 2F; Fig. S3A-H). Thus, although the *MR26F8* mice were viable into adulthood, they already had significant craniofacial abnormalities by birth. Therefore, we compared the underlying skeleton of control and *MR26F8* mice during early postnatal development using bone and cartilage staining (Fig. 2G-L; Fig. S3M-P). In the postnatal day (P) 0 wild-type skull, the bony plates of the frontal, parietal and interparietal bones had not yet met at the dorsal midline, and the coronal and lambdoid sutures were clearly visible as an overlapping region between adjacent bony plates (Fig. 2G). By contrast, in *MR26F8* mice, the sagittal and interfrontal sutures were wider, except in the region where the coronal and midline sutures should intersect (Fig. 2J). Here, there was a narrowing of the sagittal and interfrontal sutures (Fig. 2J). With respect to the lambdoid suture, the interparietal and parietal bones did not overlap to the same extent as in the controls. Most strikingly, however, the coronal suture separating the frontal and parietal bones was abnormal (Fig. 2J). In some areas, where a coronal suture would be expected, there were instead wider and irregular regions where the bones did not overlap. Such irregular fissures were a common feature in the *MR26F8* mice, whereas a typical coronal suture was never observed in this genotype. In other regions, particularly nearer the dorsal midline, there was no apparent suture and the frontal and parietal bones were fused together seamlessly. These suture defects were accompanied by decreased ossification of the ventral portions of the parietal bones (Fig. S3N). Despite the numerous defects in the cranial vault, ossification of the cranial base remained largely unaffected (Fig. S3P).

From P12 onwards, some cranial defects in the *MR26F8* mice had resolved, whereas others had materialized, including ectopic bone development in the base of the eye socket (Fig. 2J-L). With respect to the calvaria, in the P12 and adult wild-type skulls, the bony plates of the frontal, parietal and interparietal bones had met at the dorsal midline to form distinguishable sagittal and interfrontal sutures, and the coronal and lambdoid sutures were still apparent (Fig. 2H,I). Similarly, in the *MR26F8* mice, the skull bones had now met at the midline to form the sagittal and interfrontal sutures. However, craniosynostosis of the coronal suture in the mutants was now complete (Fig. 2K,L) and the lambdoid suture was now narrower than in the wild type. *MR26F8* mice also exhibited misalignment of the frontonasal region, a condition that was associated with variable defects in the interactions between the frontal, nasal and premaxillary bones, including the presence of Wormian bones, partial fusion of the frontonasal suture and a lack of interdigitation of the frontopremaxillary suture (Fig. 2K,L and data not shown).

**Fig. 3.**
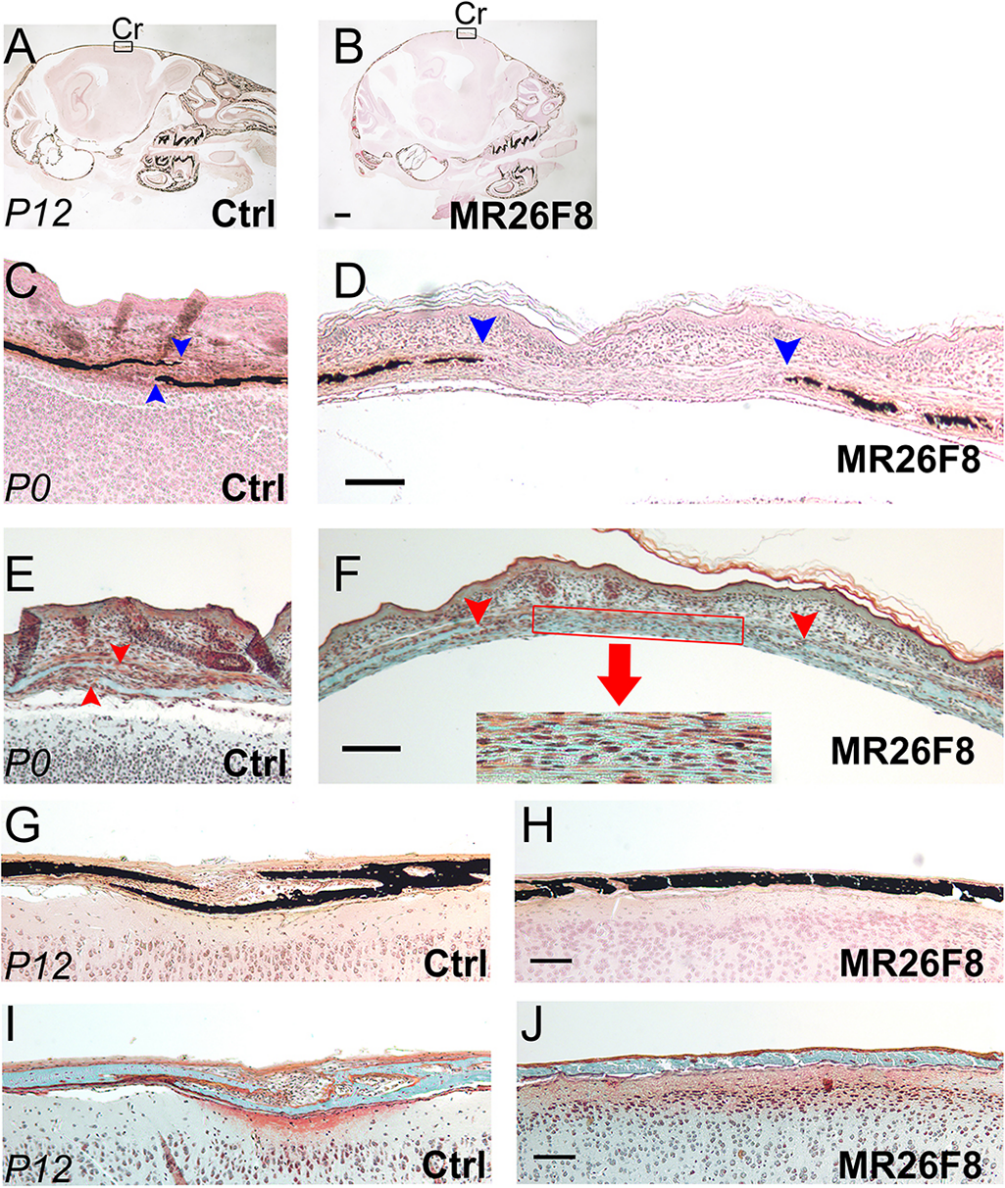
**Delayed ossification, followed by craniosynostosis, of the *MR26F8* coronal suture.** Low magnification images in A and B show the approximate position of the coronal suture (Cr) from P12 sagittal sections. Sagittal sections from P0 (C-F) and P12 (G-J) skulls for control (A,C,E,G,I) and *MR26F8* (B,D,F,H,J) pups. (A-D,G,H) von Kossa staining with mineralized bone appearing dark red/black. (E,F,I,J) Goldner's trichome staining highlighting mature (green) and immature (red) bone matrix. In the P0 sections, blue arrowheads (C,D) mark the limits of mineralization; red arrowheads (E,F) show the extent of the unmineralized mature osteoid, with a mixture of mature and immature bone matrix occurring between them in the mutant as shown in greater detail in the inset (F, red arrow). Scale bars: (B) 1 mm; (D,F,H,J) 100 µm. Aged-matched controls and mutants are at the same scale; *n*=3.

To examine the craniosynostosis of the coronal suture in more detail, we sagittally sectioned both P0 and P12 craniums and then employed von Kossa and Goldner's trichrome stains to assess mineralization and bone differentiation, respectively. The von Kossa-stained neonate samples showed that, compared with controls, the *MR26F8* mice had a wider, more open region between the mineralized bone fronts, giving the superficial appearance of a broader coronal suture (Fig. 3C,D). Goldner's trichome staining of *MR26F8* samples revealed that this wider area was composed of an unorganized combination of mature and immature bone matrix, as opposed to undifferentiated suture mesenchyme (Fig. 3F), indicating that there is abnormal or premature osteoblast differentiation in this area. By P12, all *MR26F8* mice exhibited premature fusion of the coronal suture (Fig. 3H,J) compared with the control sutures, which were not fused (Fig. 3G,I). Therefore, the coronal suture in the *MR26F8* mice displayed ectopic matrix deposition with wider separation of both the osteoid and mineralized fronts followed by mineralization of this intervening matrix and subsequent craniosynostosis. The same phenomena of relatively wide separation between the mineralized bone fronts at early stages, with subsequent narrowing compared with controls, was also observed in the *MR26F8* lambdoid sutures, although these structures did not undergo overt fusion during the time points examined (Fig. S5). In summary, moderately increased *Fgf8* expression caused cranial vault ossification defects, most notably synostosis of the coronal suture.

### Abnormal cartilage replaces intramembranous bone at higher Fgf8 levels

**Fig. 4.**
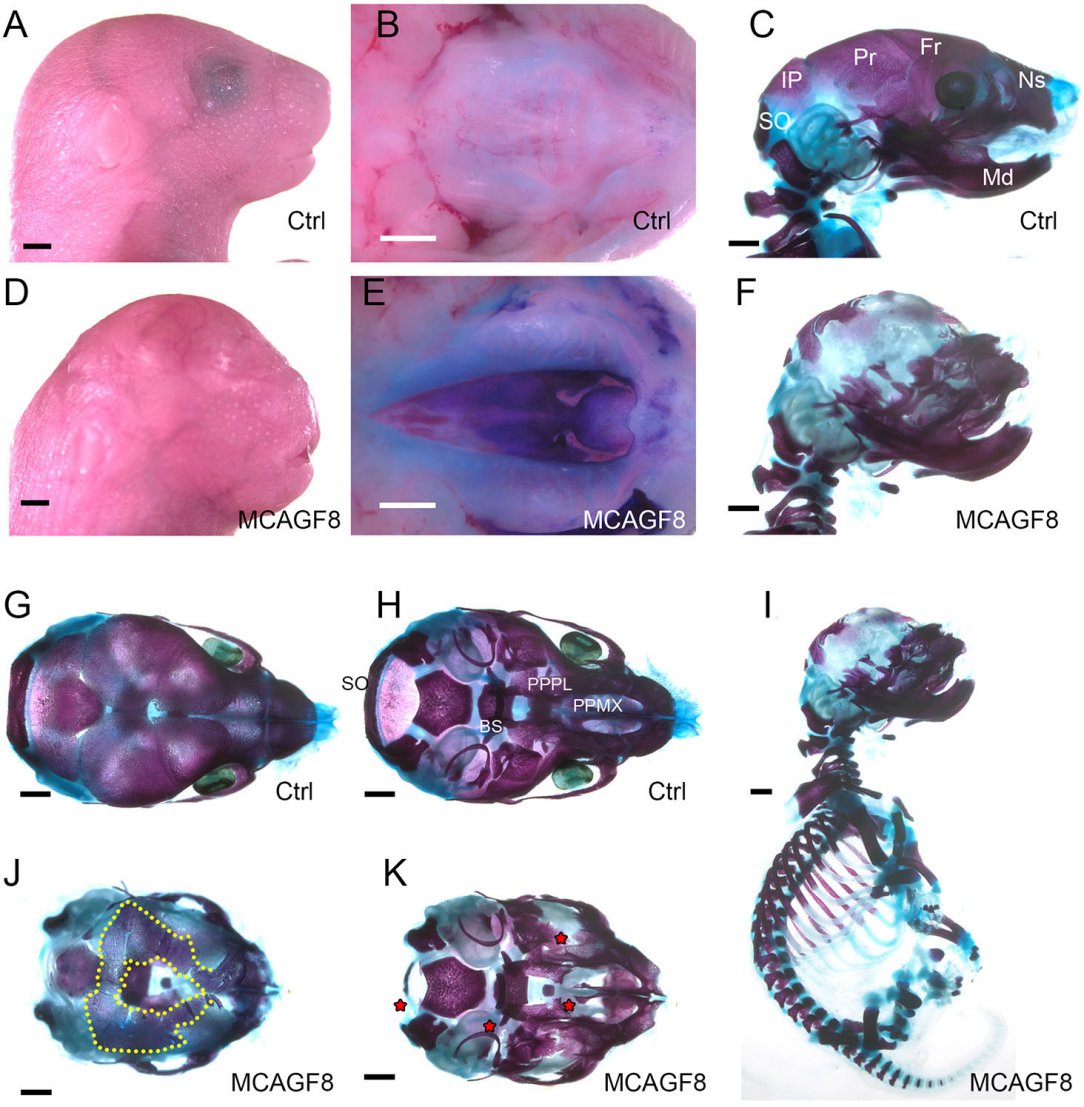
***MCAGF8* mice exhibit cranial defects including loss of ossification.** (A,B,D,E) Gross images of control (A,B) and mutant (D,E) E18.5 embryos showing lateral views of whole head (A,D) or ventral views of secondary palate after removal of lower jaw (B,E). Note, the more intense blue staining of the palate in E versus B results from an examination of skin permeability using Toluidine Blue staining. (C,F-K) Bone and cartilage staining of the control (C,G,H) and mutant (F,J,K) skulls, as well as the entire mutant body (I). (C,F,I) Lateral view; (G,J) dorsal view of the cranial vault; (H,K) ventral view of cranial base after removal of the lower jaw. (J) The yellow dotted line notes the boundary between bone and non-stained matrix formation. Red stars denote *MCAGF8* ossification gains or losses. BS, basisphenoid; Fr, frontal; IP, interparietal; Md, mandible; Ns, nasal; PPMX, palatal process of the maxilla; PPPL, palatal process of the palatine; Pr, parietal; SO, supraoccipital. Scale bars: 1 mm; *n*=15.

As with the *MR26F8* mice, the *Msx2-Cre;CAG^Fgf8b^* mice, hereafter termed *MCAGF8*, were born at normal size and Mendelian ratios. However, unlike the *MR26F8* mice, the *MCAGF8* mice did not survive beyond the first day, probably owing to altered facial shape and clefting (Fig. 4A-F). Thus, our analysis was limited to P0 and embryological time points. *MCAGF8* neonates exhibited several striking craniofacial phenotypes: a shortened snout, domed skull, cleft secondary palate and loss of the eyes. Other morphological defects included limb deformities, particularly post-axial polydactyly, and more complex fusion phenotypes (Fig. S6).

We next compared E18.5 skeletons of *MCAGF8* and control embryos using standard bone and cartilage staining. These studies revealed that *MCAGF8* mice had a surprising skeletal phenotype. Specifically, whereas the calvaria of controls were composed of bone (Fig. 4C), the bone of the *MCAGF8* calvaria was mostly replaced with a matrix that stained with neither Alcian Blue nor Alizarin Red, but was still durable enough to persist through a staining protocol that degrades soft tissue. By comparison, the endochondral bones and cartilage throughout the rest of the body stained normally (Fig. 4I). Dorsal views of the skull revealed a ring of bone surrounding the midline, with non-stained matrix radiating ventrally from there (Fig. 4J). Any sutures, if present, were difficult to identify owing to the prevalence of the non-stained matrix. The skeletal defect was not uniformly distributed throughout the calvaria, as there was a greater amount of ossification in the region where the parietal bones would normally form (Fig. 4F,J). One possible explanation for the presence of bone in this region is that there was reduced activity of *Msx2-Cre* in this location as shown by fate mapping (Fig. S2C). Although the primary affected area was the cranial vault, there were also defects within other components of the craniofacial skeleton (Fig. 4H,K). Several skeletal elements were smaller, including the palatal process of the maxilla and palatal process of the palatine (Fig. 4K), consistent with the cleft secondary palate (Fig. 4E). There was also an overall shortening of the snout and its associated skeletal elements in the mutants (Fig. 4F,J,K), as well as a reduced supraoccipital bone (Fig. 4J,K). Additionally, like the *MR26F8* mice, the *MCAGF8* mice have ectopic bone growth within the eye sockets (Fig. 4J,K). Overall, the *MCAGF8* mice exhibit several abnormalities, including craniofacial patterning defects, loss of the eye, and limb defects. Most strikingly, however, was the replacement of the cranial vault bones with an abnormal non-stained matrix.

**Fig. 5.**
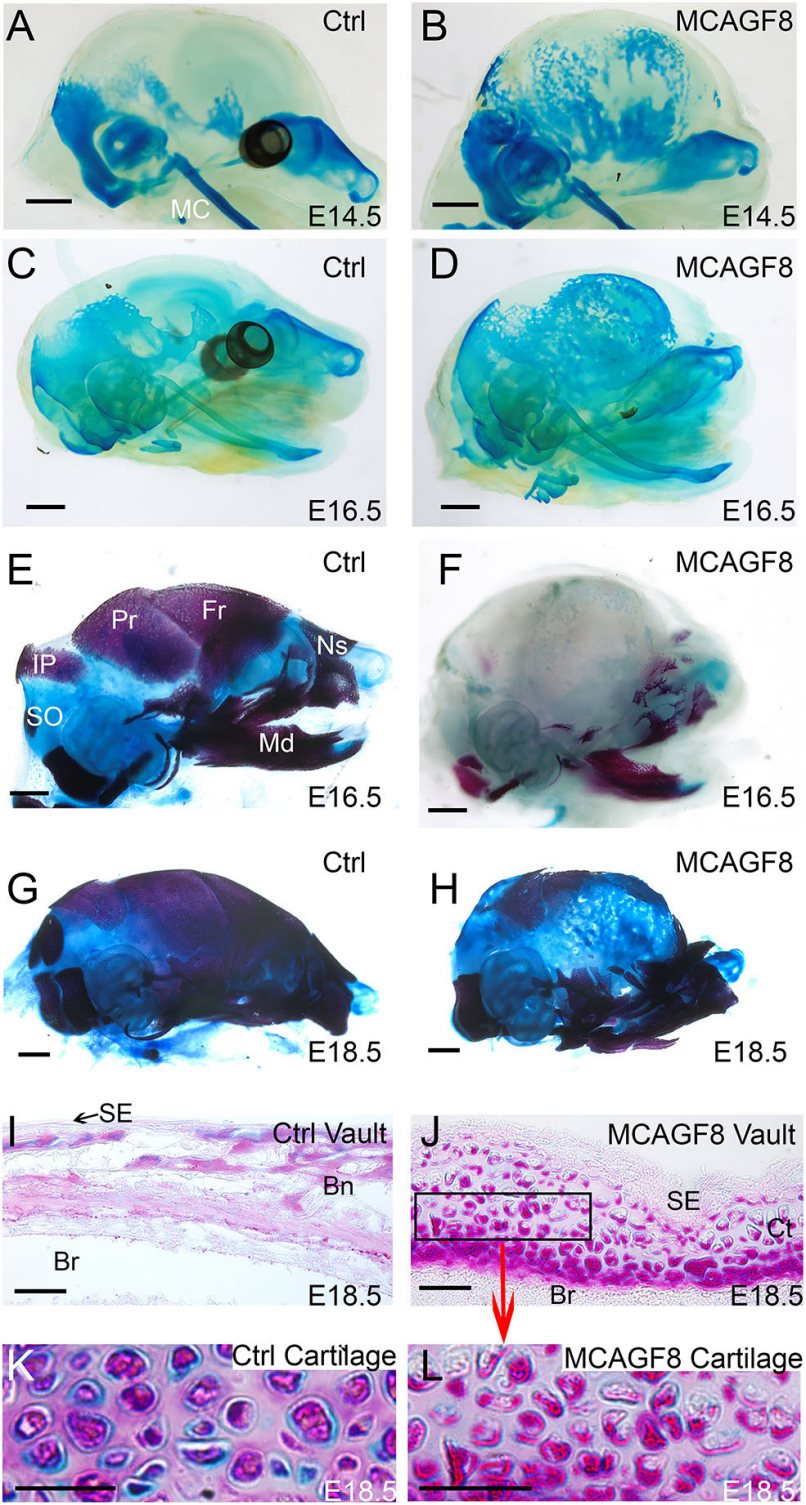
**Cartilage replaces bone in the *MCAGF8* cranial vault.** (A-H) Lateral views of control (A,C,E,G) and *MCAGF8* (B,D,F,H) skulls. (A-D) Bouin's fixed skulls stained for cartilage (Alcian Blue) at E14.5 (A,B) and E16.5 (C,D). (E,F) Bone and cartilage staining with Alizarin Red (bone) and Alcian Blue (cartilage) at E16.5. (G,H) Bone and cartilage staining with Alizarin Red, Alcian Blue and Toluidine Blue (cartilage) at E18.5. (I-L) Alcian Blue+PAS stain of coronal sections from (I) control cranial vault, (J,L) *MCAGF8* lateral cranial vault, and (K) control cranial base cartilage (primordium of the presphenoid bone). Acidic mucins stain blue, neutral mucins stain magenta. Bn, bone; Br, brain; Ct, cartilage; Fr, frontal; IP, interparietal; MC, Meckel's cartilage; Md, mandible; Ns, nasal bone; Pr, parietal; SE, surface epithelium; SO, supraoccipital bone. Scale bars: (A-H) 1 mm, (I-L) 40 µm; A-F, *n*=5; G-K, *n*=3.

To determine the origins and composition of this unusual matrix, we examined crucial stages in embryological development when bone and cartilage are being formed. At E14.5, prior to extensive bone mineralization, we utilized a cartilage-staining protocol with Bouin's fixative and Alcian Blue (Fig. 5A,B). This staining regimen was also used at E16.5 alongside a standard protocol that uses Alcian Blue and Alizarin Red to stain cartilage and bone, respectively (Fig. 5C-F). Finally, the standard protocol was used to stain E18.5 samples (Fig. 5G,H). Differences between the controls and *MCAGF8* mice were first apparent at E14.5 (Fig. 5A,B). In the E14.5 controls, cartilage was forming at the site of the jaw, snout, cranial base and otic capsule (Fig. 5A). The *MCAGF8* mice also had cartilage in these locations but further displayed ectopic cartilage throughout almost the entire cranial vault, which was still apparent at E16.5 (Fig. 5D). Surprisingly, however, this same matrix did not stain at E16.5 or E18.5 when Alcian Blue and Alizarin Red were used for skeletal staining, possibly due to the difference in pH between the two staining solutions (Fig. 5F and Fig. 4F, respectively). Notably, however, this matrix did stain when we employed Toluidine Blue, another cartilage stain, alongside Alizarin Red and Alcian Blue on E18.5 preparations (Fig. 5G,H, Fig. S7F,G). Histological analysis of sectioned material confirmed that there was a loss of both ossification and mineralization in the *MCAGF8* skulls compared with controls (Fig. 5I,J, Fig. S7A,C). Instead, there was development of tissue comprised of nuclei within lacunae surrounded by a matrix that stains strongly with Toluidine Blue, typical of hyaline cartilage (Fig. S7D,E). We next used further histological analysis to compare this skull cartilage with control cartilage from the cranial base to probe for differences that might reflect the unusual staining properties of this ectopic tissue in the *MCAGF8* mice. A notable difference in matrix composition became apparent when a dual Alcian Blue periodic acid-Schiff (PAS) staining protocol was employed (Fig. 5J-L). Specifically, *MCAGF8* cartilage stained weaker with Alcian Blue and stronger with PAS, indicating that this cartilage has less acidic mucins (i.e. glycosaminoglycans) and more neutral mucins (i.e. polysaccharides and mucosubstances) than control samples. Thus, overall, the high *Fgf8b* levels cause cranial chondrogenesis to be favored over osteogenesis in the *MCAGF8* mice. However, the resulting cartilage is abnormal with an altered ratio of neutral to acidic mucins.

### Differential effects of increased *Fgf8* transcript levels on intramembranous and endochondral bones

Although the expression pattern of the *Msx2-Cre* transgene was suitable for studying development of the calvaria, it was not efficient at directing *Fgf8b* to other elements of the craniofacial skeleton that form by intramembranous ossification, such as the dentary bone. At the same time, the expression of *Msx2-Cre* in the limb ectoderm did not appear to affect ossification of endochondral bones in the limb, whereas the supraoccipital bone in the skull, which forms via endochondral ossification, was greatly reduced in the *MCAGF8* mice (Fig. 4). Thus, to ascertain how increased *Fgf8b* expression affects both types of ossification more globally throughout the embryo, we used the two new *Fgf8b* alleles alongside osteocalcin-Cre (*OC-Cre*). *OC-Cre* is expressed in osteoblasts in both endochondral and intramembranous-forming bone beginning around E14.5 (Zhang et al., 2002) (Fig. S8). Once again, the increase in *Fgf8b* expression resulted in developmental defects, with the resulting *OR26F8* (*OC-Cre;R26^Fgf8b^*) and *OCAGF8* (*OC-Cre;CAG^Fgf8b^*) mice being readily distinguishable from controls by gross morphology at E18.5, owing to shortened mandibles and limbs (Figs S9 and S10, and data not shown).

**Fig. 6.**
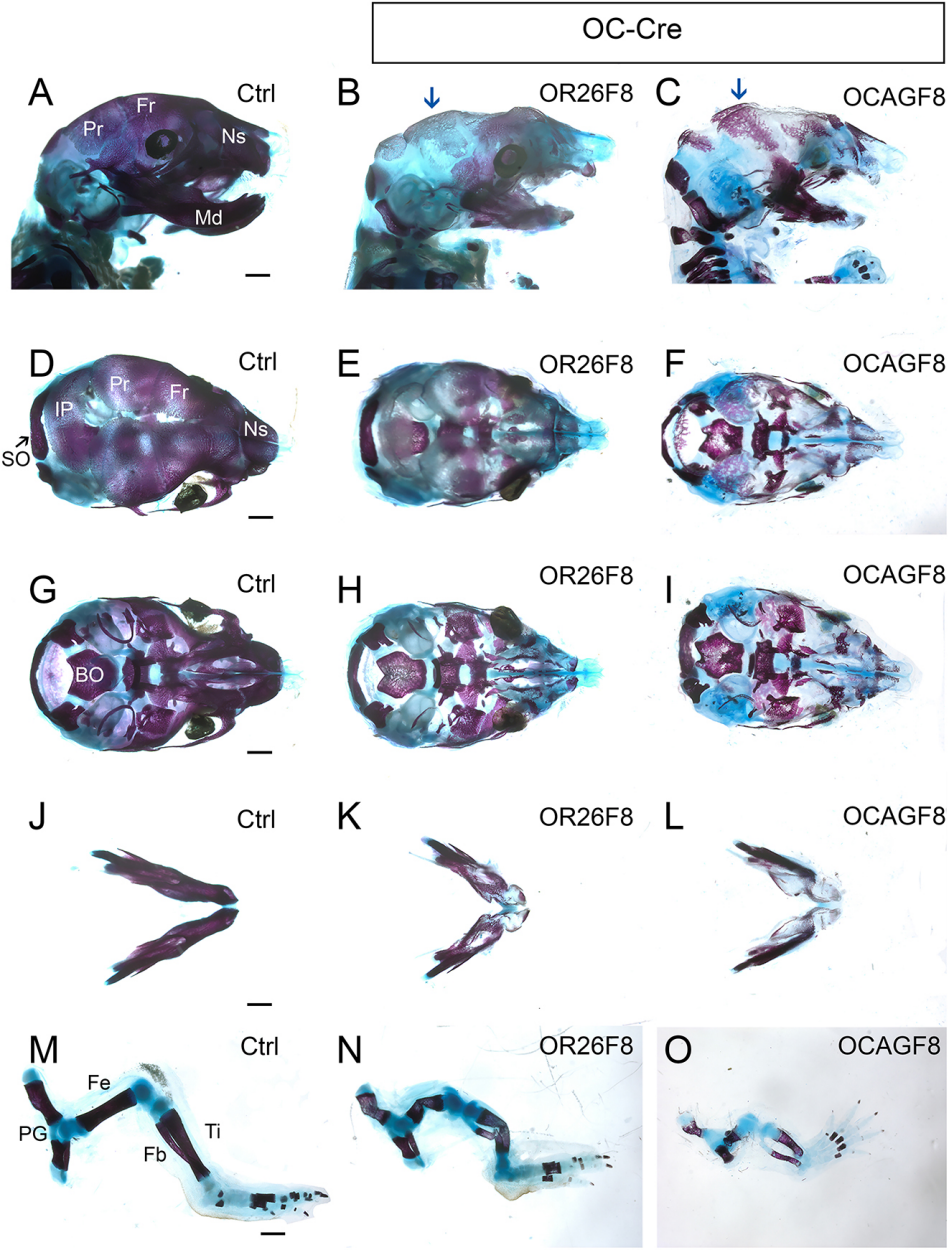
**Intramembranous ossification is more severely affected by increased *Fgf8b* expression than endochondral ossification.** (A-O) Bone and cartilage staining of E18.5 control (A,D,G,J,M), *OR26F8* (B,E,H,K,N) and *OCAGF8* (C,F,I,L,O) samples. (A-C) Lateral view of head, (D-F) dorsal view of cranial vault, (G-I) ventral view of cranial base after mandible removal, (J-L) dorsal view of mandibles, (M-O) hindlimbs. Blue arrows indicate parietal bones. BO, basioccipital; Fb, fibula; Fe, femur; Fr, frontal; IP, interparietal; Md, mandible; Ns, nasal; PG, pelvic girdle; Pr, parietal; SO, supraoccipital; Ti, tibia. Scale bars: 1 mm. Controls, *OR26F8* and *OCAGF8* samples are shown at the same magnification; *n*=5.

Both *OR26F8* and *OCAGF8* mice had a number of similar skeletal defects, which were more severe in the latter (Fig. 6). The calvaria were mostly replaced by an unstained cartilage matrix, in common with *Msx2-Cre*-based mice, although there were some notable differences in the most affected regions. Thus, whereas *Msx2-Cre*-based mice had some ossification surrounding the midline sutures, this top-most region of the cranial vault consisted solely of non-stained matrix in the *OC-Cre* mutants (Fig. 6E,F, Fig. S11). By contrast, there was more lateral ossification in both *OC-Cre* mutants (Fig. 6B,C) than there was in the *MCAGF8* mice (Fig. 4F), probably owing to the later initiation of *OC-Cre* expression. One notable similarity, however, was that the parietal bones had the most ossification and were least affected when either *Msx2-Cre* or *OC-Cre* were employed. With respect to additional craniofacial bones that form via intramembranous ossification, both *OR26F8* and *OCAGF8* mandibles were shorter than the controls and did not ossify normally, but instead were partially replaced by non-stained matrix (Fig. 6J-L). Similarly, the bones of the maxilla and palate had severely decreased ossification and were mostly replaced by non-stained matrix, particularly in the *OCAGF8* mice (Fig. 6D-I). By contrast, for bones formed via endochondral ossification we did not observe a replacement of bone by cartilage or by an abnormal unstained cartilage matrix (Fig. 6G-I,M-O). The disparity between the formation of the two types of bone in the mutants was also evident when alkaline phosphatase staining was used to examine bone differentiation (Fig. S12). Nevertheless, many endochondral bones, such as the basioccipital in the cranial base, were less mineralized than controls, as judged by Alizarin Red staining (Fig. 6G-I), and there were also size and shape differences, most notably for the limb skeleton. Therefore, increased *Fgf8b* expression, derived from either adjacent tissues or from within the developing bone itself, significantly affected intramembranous ossification and caused additional, yet variable, defects with respect to endochondral bone formation.

### Dysregulation of bone- and cartilage-related genes in the *MCAGF8* skull

**Fig. 7.**
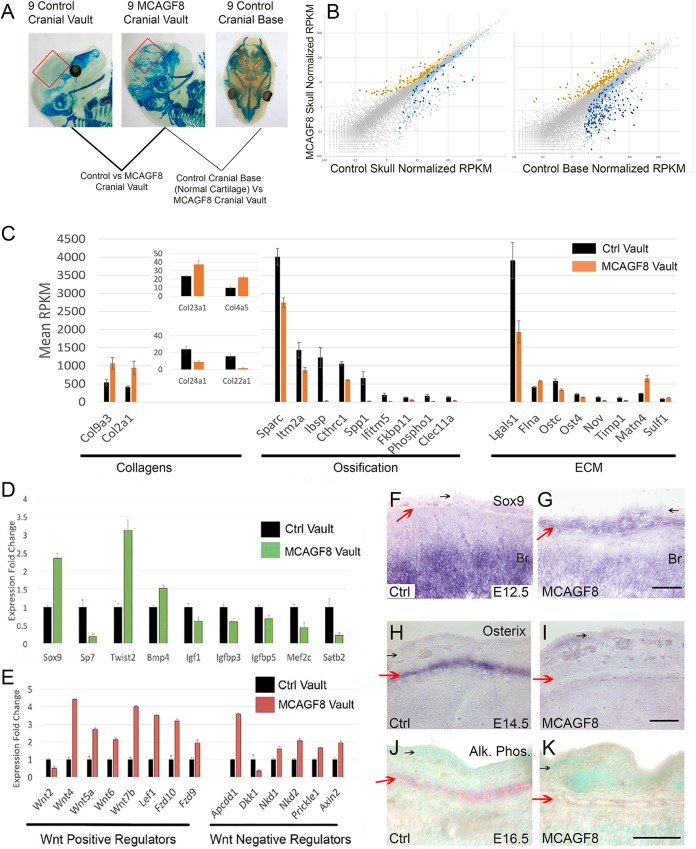
***MCAGF8* cranial vault differentiation shifts from osteogenic to chondrogenic.** (A) Schematic of RNAseq experimental design. E14.5 tissue was removed from control (left) and *MCAGF8* (center) cranial regions, outlined by the red boxes, after skin removal and avoiding underlying brain tissue. Control cranial base cartilage was removed from areas shown by red dots (right). (B) Scatterplot depicting average reads per kilobase of transcript per million mapped reads (RPKM) values. Colored points represent transcripts that are statistically significant between the groups: *MCAGF8* cranial vault (*y*-axis), and either control cranial vault (*x*-axis, left) or control cranial base (*x*-axis, right). Yellow represents genes upregulated in *MCAGF8* compared with the control (light yellow=*P*<0.05, dark yellow=*P*<0.05 and q<0.1). Blue represents genes down­regulated in *MCAGF8* compared with the control (light blue=*P*<0.05, dark blue=*P*<0.05 and q<0.1). (C) Histograms plotting normalized RPKM values of genes associated with collagen (left), cartilage/bone differentiation (middle) and ECM (right) that are significantly dysregulated in *MCAGF8* cranial vaults (orange) as compared with control vaults (black). Error bars represent standard error. (D,E) Histograms plotting expression fold change in *MCAGF8* cranial vault samples as compared with controls: control values are normalized to 1 (black bars), whereas mutant samples are represented by green or red, respectively, for genes involved in differentiation (D) or Wnt signaling (E). Error bars represent standard error. (F-I) RNA *in situ* hybridization in the cranial vault for expression (purple) of *Sox9* at E12.5 (F,G) or *Osterix/Sp7* at E14.5 (H,I) for control (F,H) and *MCAGF8* (G,I) embryos. (J,K) Alkaline phosphatase activity (red) in the cranial vault of E16.5 control (J) and *MCAGF8* (K) embryos. The cranial vault (red arrows) and surface epithelium (black arrows) are shown. Br, brain. Scale bars: 60 µm; *n*=3.

Given the differential effect of increased *Fgf8b* levels on the process of intramembranous ossification, we next explored the basis of this phenomenon by comparing gene expression in the developing cranial vault between *MCAGF8* mice and controls using RNA sequencing (RNAseq; Fig. 7A). We dissected cranial mesenchymal tissue between the skin and brain from the controls and *MCAGF8* mutants at E14.5, when cranial bone development is initiating. We also sampled tissue from E14.5 control cranial base as a baseline for hyaline cartilage gene signatures. The results from these comparisons are presented in Tables S1 and S2, respectively, and summarized in Fig. 7B. Note that, consistent with the removal of the skin where the *MCAGF8* allele was expressed before RNAseq analysis, *Fgf8* was not differentially expressed between *MCAGF8* and control skull tissue (Table S1). By contrast, both *Dusp6* and *Spry4*, which are both reporters and feedback regulators of FGF signaling, were upregulated in the *MCAGF8* mesenchyme. Additionally, *Htra1*, which directly cleaves and deactivates FGF8 in the extracellular environment, was upregulated in the mutants, potentially indicative of attempted feedback regulation of FGF8 signaling. These observations are consistent with FGF8 signaling from the ectoderm affecting gene expression in the underlying mesenchyme.

Next, to identify important categories of differentially expressed genes between the control and *MCAGF8* cranial vault samples, we used DAVID functional annotation clustering and functional annotation charting to analyze these data sets (Huang et al., 2009b). Comparison of *MCAGF8* and control cranial vaults yielded differentially expressed genes annotated to categories including bone development, ossification, osteoblast differentiation, regulators of bone mineralization, Wnt signaling and categories likely to reflect the shift to cartilage differentiation; for example, genes encoding proteins involved in formation of the extracellular matrix, glycoproteins, glycosylation and disulfide bonds (Table S1). On the basis of this DAVID analysis, we next focused on the expression of transcripts encoding major structural components of bone and cartilage in the calvaria samples of controls and *MCAGF8* mice. These analyses showed *MCAGF8* mutants exhibited a major increase in the expression of *Col2a1* and *Col9a3*, two collagens typically found together in hyaline cartilage (Mayne, 1989) (Fig. 7C, left). Conversely, *Col24a1*, a gene involved in osteoblast differentiation and bone formation (Koch et al., 2003), was downregulated (Fig. 7C, Table S1). Two additional genes involved in bone formation, *Ibsp* and *Spp1*, were downregulated by >30-fold in the *MCAGF8* cranial vault tissue, whereas others associated with this process were decreased to a lesser extent, including *Sparc*, *Ifitm5*, *Fkbp11*, *Phospho1*, *Itm2a*, *Cthrc1* and *Clec11a* (Fig. 7C, Table S1). Together, these findings indicate that the mutants favor a cartilage rather than a bone program of gene expression. However, the effects of ectopic *Fgf8b* on the transcription of genes involved in bone development were not uniform (Fig. 7C), implying that specific aspects of osteogenesis were more severely affected. Alongside these structural components, genes responsible for collagen processing and extracellular matrix (ECM) formation and modification, including *Sulf1*, *Ost4* and *Ostc*, were also dysregulated in the *MCAGF8* skulls (Fig. 7C, right; Table S1). The aberrant expression of genes involved in collagen modification, as well as the changes in genes encoding enzymes that alter glycosylation and sulfation of ECM proteins, provides a possible explanation for the altered staining properties of the cartilage found in the *MCAGF8* cranial vaults.

Next, we determined whether there were gene expression differences between the cartilage of the *MCAGF8* skull versus control cranial base cartilage (Table S2). More genes were found to be dysregulated in this comparison, and dysregulated at higher levels, than between the control and mutant cranial vault samples (Fig. 7B). The most striking observation was that *Col10a1*, which is crucial for endochondral bone formation (Gu et al., 2014), was expressed at >100-fold lower levels in the *MCAGF8* cranial vaults (Table S2). Several additional genes involved in endochondral ossification were also differentially expressed between these two samples, with higher levels in the control cranial base compared with the *MCAGF8* cranial vault cartilage. These genes included *Mmp13* (>10-fold), *3110079O15Rik* /*Snorc* (>10-fold), *Ihh* (∼10-fold), *Spp1* (>5-fold) and *Ibsp* (>5-fold) (Table S2). Thus, although differentiation in the cranial vault favors a chondrogenic versus osteogenic trajectory in the presence of upregulated *Fgf8b* signaling in *MCAGF8* mice, it is not undergoing normal endochondral ossification and displays distinct histochemical properties from other cartilage in the body (as shown in Fig. 5).

### *MCAGF8* mutants have impaired differentiation and display dysregulated Wnt signaling

We next examined the balance between regulators of chondrogenesis and osteogenesis in the control and *MCAGF8* skulls to determine how *Fgf8b* was exerting its mechanistic effects. These analyses indicated that several regulators of cartilage/bone differentiation were dysregulated in the *MCAGF8* cranial vaults (Fig. 7D). First, there was a shift towards the expression of transcription factors that are linked with a chondrocyte cell fate and inhibit ossification. In particular, *Sox9*, which stimulates chondrocyte differentiation (Akiyama et al., 2002; Lefebvre et al., 1997; Lefebvre and de Crombrugghe, 1998), *Twist2*, an inhibitor of osteoblast differentiation (Bialek et al., 2004; Lai et al., 2011; Lee et al., 1999), and *Irx1* and *Irx5*, markers of immature chondrocytes, were all upregulated (Fig. 7D, Table S1). Conversely, mRNAs for *Sp7*/*Osterix*, *Mef2c* and *Satb2*, transcriptional regulators that stimulate bone differentiation, were downregulated. Comparing the *MCAGF8* skull vault with the control cranial base indicated that the latter was more similar to the control skull vault with respect to the expression profiles of these same transcription factors (Table S2). This finding was again consistent with the cranial base initiating endochondral bone formation, whereas the *MCAGF8* skull vault has an expression profile that favors cartilage over bone, reinforcing the difference between these two types of cartilage. We also examined the expression of *Sox9* and *Sp7* using *in situ* hybridization in control and *MCAGF8* skulls (Fig. 7F-I). These results demonstrated that *Sox9* was upregulated in the *MCAGF8* skulls as early as E12.5, before overt ossification occurs, and this was mirrored by a later decrease in *Sp7* expression at E14.5. Next, to determine whether dysregulation of osteoblast regulatory genes correlated with impaired differentiation, we examined alkaline phosphatase activity, a marker of bone differentiation, at E16.5 using a liquid Fast-Red staining protocol. The *MCAGF8* cranial vaults had very little alkaline phosphatase activity (Fig. 7K) compared with controls (Fig. 7J). Together, these data indicate that *Fgf8b*-induced changes early in the differentiation process are probably responsible for favoring chondrogenesis over osteogenesis in the skull vault.

It has been previously shown that loss of β-catenin protein in the skull can switch development of the calvaria from bone to cartilage (Goodnough et al., 2012; Tran et al., 2010). Wnt signaling was also highlighted by the DAVID functional annotation clustering in the current analysis, where it occurred as the fifth annotation cluster with an enrichment score of 4.2 (Table S1). Therefore, we next investigated Wnt signaling pathway aberrations in the *MCAGF8* skull. These analyses revealed that multiple inhibitors and negative regulators of Wnt signaling were upregulated in the *MCAGF8* cranial vaults, including *Apcdd1*, *Axin2*, *Kremen2*, *Nkd1*, *Nkd2* and *Prickle1* (Fig. 7E and Table S1). At the same time, several genes that positively regulate Wnt signaling were upregulated in the *MCAGF8* cranial vaults, including those encoding the Wnt ligands 4, 5a, 6 and 7b. Additionally, members of the canonical Wnt signaling pathway, including frizzled receptors (*Fzd9*, *Fzd10*) and the transcription factor *Lef1* (Fig. 7E), were also upregulated. By contrast, only one Wnt ligand, *Wnt2*, and one Wnt pathway inhibitor, *Dkk1*, were significantly downregulated. In addition to the striking effect of *Fgf8b* overexpression on Wnt signaling, there were also clear effects on other signaling pathways, notably the HH, insulin-like growth factor and transforming growth factor β/bone morphogenic protein (TGF-β/BMP) pathways, which might all be predicted to alter bone growth and development (Table S1).

### Reducing *Axin2* gene dosage partially rescues the cranial skeleton phenotype

**Fig. 8.**
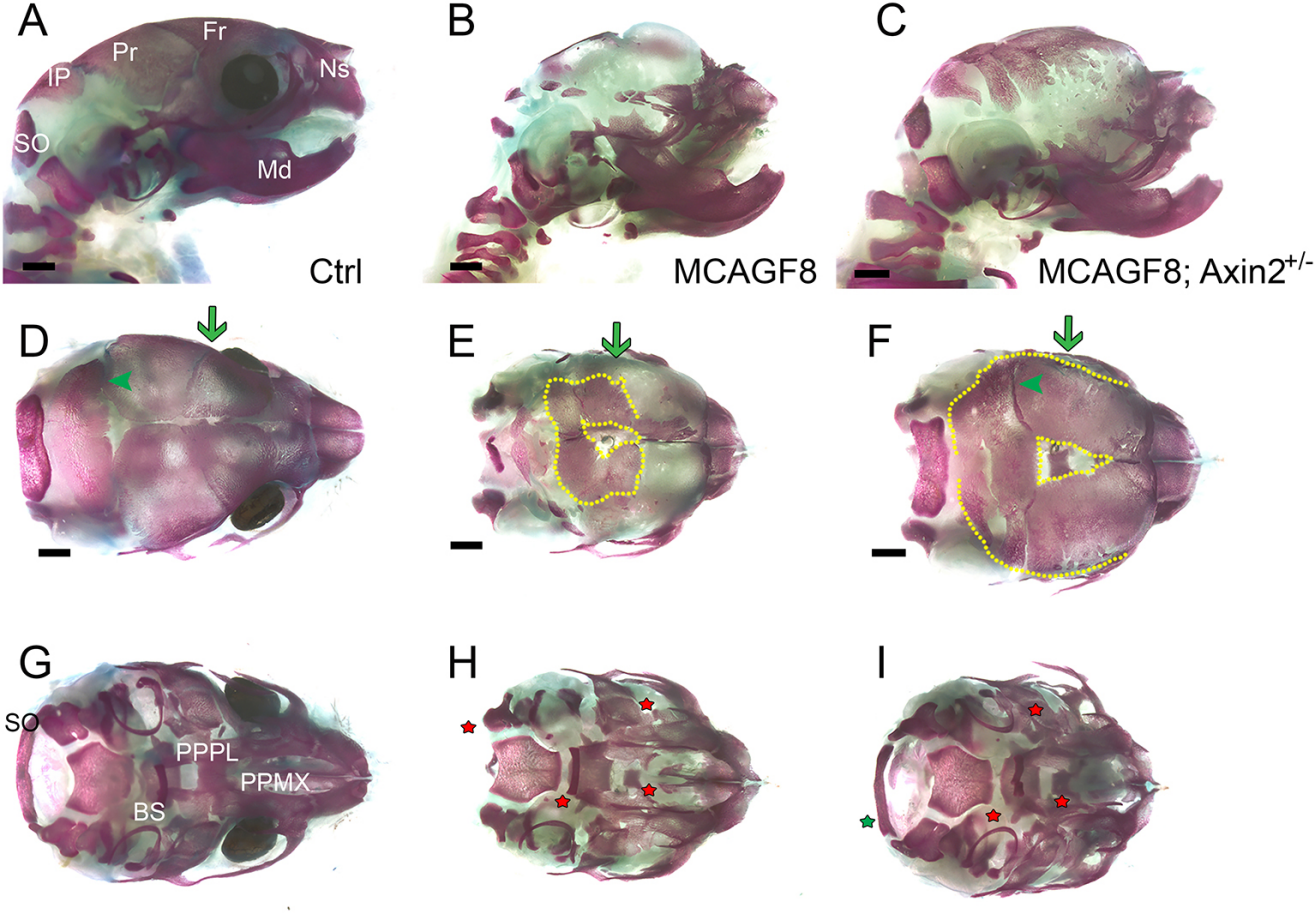
**Reduced *Axin2* gene dosage improves *MCAGF8* cranial vault ossification.** (A-I) Bone and cartilage staining of P0 control (A,D,G), *MCAGF8* (B,E,H) and *MCAGF8;Axin2^lacz+/−^* mutants (C,F,I); *n*=5 per genotype. (A-C) A lateral view, (D-F) a dorsal view of the cranial vault and (G-I) the cranial base. The boundary between bone and non-stained matrix (yellow dashed line), the presence or absence of the coronal suture (green arrow) and lambdoid suture (green arrowhead) are shown. Red stars denote regions of the cranial base that differ from the control; the green star in (I) shows the rescue of the supraoccipital bone. BS, basisphenoid; Fr, frontal bone; IP, interparietal bone; Md, mandible; Ns, nasal bone; PPPL, palatal process of the palatine; PPMX, palatal process of the maxilla; Pr, parietal bone; SO, supraoccipital. Scale bars: 1 mm; *n*=5.

Although the expression of many signaling genes were altered in the *MCAGF8* model, the RNAseq data alone do not provide a clear indication of how these alterations could affect intramembranous ossification. However, given the many alterations in the Wnt signaling pathway, as well as the connections between β-catenin and intramembranous ossification, we further probed the interaction between FGF and Wnt signaling *in vivo*. Specifically, we bred an *Axin2^lacZ^* allele into the *MCAGF8* background (Fig. 8). *Axin2* expression is stimulated by the canonical Wnt signaling pathway and also acts as a feedback regulator of the pathway through the degradation of β-catenin (Jho et al., 2002). The *Axin2^lacZ^* allele replaces the normal gene with a nuclear localization signal (NLS)-*lacZ* transgene (Lustig et al., 2002) so that normal *Axin2* expression decreases in heterozygotes, theoretically increasing Wnt signaling. Initial gross morphological analysis of the *MCAGF8;Axin2^+/−^* mice indicated that several *MCAGF8* phenotypes still persisted, including the missing eye, the shortened snout and the cleft palate (data not shown). However, subsequent skeletal staining indicated a clear difference in skull ossification between the two models. Thus, in contrast to the *MCAGF8* skulls, which had some ossification near the midline suture with non-stained matrix throughout most of the skull (Fig. 8B,E), the *MCAGF8;Axin2^+/−^* mice all showed an expansion of ossification midway down the skull, with non-stained matrix only on the lateral part of the skull, nearest the cranial base (Fig. 8C,F). The expansion of bone development in *MCAGF8;Axin2^+/−^* mice also led to the reappearance of the lambdoid suture (Fig. 8F), a feature not apparent in the *MCAGF8* mice owing to the absence of bone (Fig. 8E). However, increased bone development did not lead to the rescue of all sutures, as the *MCAGF8;Axin2^+/−^* mice displayed craniosynostosis of the coronal suture (Fig. 8F). Indeed, the suture pattern in the *MCAGF8;Axin2^+/−^* mice, with an open lambdoid suture and craniosynostosis of the coronal suture, was similar to that of the *MR26F8* mice (Fig. 2). Interestingly, the reduced *Axin2* gene dosage also rescued the hypoplasia of the supraoccipital bone seen in *MCAGF8* mice, but consistent with the gross morphology it did not alter the skeletal defects associated with the eye, snout and secondary palate. Thus, increasing Wnt signaling by reducing *Axin2* gene dosage partially rescued the shift from osteogenesis to chondrogenesis in the skull vault of *MCAGF8* mice, but did not rescue the coronal craniosynostosis or craniofacial shape phenotypes.

## DISCUSSION

Craniosynostosis is a relatively common human craniofacial defect that frequently involves mutations in the FGF signaling pathway, and in particular in the FGF receptors. Here, we have probed this pathway further by manipulating the dosage of an FGF ligand during mammalian embryonic development. Our characterization of how increased *Fgf8b* expression affects skeletal formation, particularly within the cranial vault, yields several interesting and previously undocumented findings. First, the cranial vault has a dose-dependent response to FGF8 signaling, with moderate levels of *Fgf8b* overexpression leading to coronal craniosynostosis and high levels of *Fgf8b* overexpression leading to ectopic cartilage formation. Second, a balance between FGF and Wnt signaling is crucial for driving the decision between an osteoblast or chondrocyte cell fate *in vivo* for the mesenchymal cells of the calvaria that would normally form bone through intramembranous ossification. Third, the effect of ectopic *Fgf8b* on craniofacial development is partially dependent on the timing and location of expression. Finally, *Fgf8b* overexpression affected both intramembranous and endochondral bone development, although the effects were generally more severe for the former skeletal elements.

Cranial suture patency is normally maintained through a delicate balance of proliferation and differentiation. Moderate overexpression of *Fgf8* in the cranial ectoderm of *MR26F8* mice affects this balance, resulting in coronal craniosynostosis and narrowing of the lambdoid suture. However, premature suture closure was not simply caused by accelerated ossification at the sutures. Instead, the coronal and lambdoid sutures were wider at birth than in the controls. However, by P12, the coronal suture had completely fused and the lambdoid suture was narrower than in the controls. Therefore, in this model there is a pattern of slower bone maturation accompanied by a failure to develop appropriate suture architecture that subsequently results in overgrowth and craniosynostosis. Notably, such delayed ossification followed by catch-up growth and subsequent obliteration of the sutures has also been observed in two mouse models with either loss or gain of function alterations in *FGFR2* (Eswarakumar et al., 2002; Mai et al., 2010). In fact, the majority of mouse craniosynostosis models resulting from FGF signaling aberrations have been generated using activating mutations in FGF receptors, particularly *Fgfr2* and *Fgfr3* (Flaherty et al., 2016; Su et al., 2014). Such mutations can cause ligand-independent dimerization and activation of the receptors with downstream intracellular signaling consequences dependent on the position and nature of the mutation within the gene (Burke et al., 1998; Monsonego-Ornan et al., 2000; Neilson and Friesel, 1996; Sarabipour and Hristova, 2016). Alternatively, some FGFR-activating mutations, including ones that cause Apert syndrome, do not lead to ligand-independent receptor binding, but instead cause increased affinity for different FGF ligands (Anderson et al., 1998). This is striking as patients with Apert syndrome, as well as those with Crouzon and Pfeiffer syndrome, often exhibit craniofacial phenotypes similar to those found in our mouse model of moderate *Fgf8b* overexpression: a characteristic appearance that includes a shortened face and premature fusion of the coronal sutures. Additionally, as in our model, patients with Apert syndrome frequently exhibit other symptoms such as cleft palate and/or limb defects, including syndactyly as well as occasional polydactyly (Katsianou et al., 2016; Ko, 2016).

Using a second allele that produces higher levels of *Fgf8b* expression, an unexpected phenotype emerged in which extensive cartilage formation replaced ossification across the cranial vault. Moreover, this ectopic hyaline cartilage did not have a gene expression profile indicative of control tissue fated to undergo endochondral ossification, but instead had several unusual properties. In particular, the cartilage extracellular matrix had an abnormal balance between acidic and basic mucins. We postulate that this switch might be a feedback control mechanism to alter the ability of the extracellular milieu to interact with the unusually high levels of *Fgf8b* in our model, as FGFs normally require heparan sulfate proteoglycans for optimal interaction with the FGFRs (Ornitz, 2000).

The extensive switch from osteogenesis to an abnormal type of chondrogenesis in the *MCAGF8* calvaria is a novel phenotype associated with altered FGF signaling. Previous studies using a transgene to express *Fgf9* in the cranial mesenchyme also induced extensive cartilage formation in the cranial vault, particularly for the region normally occupied by the parietal bones, but in this instance the cartilage was eventually replaced by bone (Govindarajan and Overbeek, 2006). We suspect that one reason for the difference between the *MCAGF8* strain and the *Fgf9* transgenic strain is that increased levels of *Fgf9* only occur transiently in this latter system potentially allowing osteogenesis at later stages, although there might be additional differences related to the FGF ligand used in each model. By contrast, once activated by Cre recombinase, the *MCAGF8* strain will continue to express ectopic *Fgf8b* constitutively, and we postulate that this maintains the abnormal cartilage phenotype and prevents bone formation. Because the *MCAGF8* mice die perinatally, we cannot rule out the possibility that *Fgf8*-induced cartilage might eventually undergo delayed endochondral ossification. However, we do not favor this possibility as in a related model system, in which *Fgf8b* is expressed only within the frontonasal prominence, the white matrix is never replaced by bone even though these mice survive into late adulthood (Fig. S13).

In addition to the aforementioned *Fgf9* transgenic mice, previous studies have shown that more limited cartilage formation can also occur in mice expressing certain *Fgfr2* mutations. In particular, mice homozygous for an activating mutation in FGFR2 (W290R), mimicking human Crouzon syndrome, exhibit thickened cartilage underlying the cranial bones (Mai et al., 2010). Similarly, in a mouse model for Apert syndrome, a heterozygous activating mutation in FGFR2 (S252W) leads to coronal synostosis and ectopic cartilage at the sagittal suture (Wang et al., 2005). Beyond the FGF signaling pathway, ectopic cartilage formation has also been observed as a consequence of aberrations in Wnt signaling (Day et al., 2005; Goodnough et al., 2012; Hill et al., 2005; Tran et al., 2010). Specifically, removal of β-catenin from cranial bone progenitors results in near-complete transformation of the skull bones to cartilage: a phenotype that is more akin to the *MCAGF8* mutant than that seen with the above-mentioned W290R and S252W *Fgfr2* alleles.

In support of this latter observation, gene expression analysis of *MCAGF8* skulls revealed dysregulation of WNT signaling in the *Fgf8b*-induced cartilage. The alterations in the Wnt pathway are accompanied by an upregulation of chondrogenic differentiation markers during a period that would normally reflect early bone development. Furthermore, expression of an inhibitor of osteoblast differentiation, *Twist2*, was also increased in *MCAGF8* calvaria concomitant with reduced expression of osteogenic markers and genes crucial for osteoblast differentiation. Together, these processes might shift the fate of mesenchymal stem cells from an osteogenic to chondrogenic potential. Alternatively, the ectopic *Fgf8b* might block a set of cells that would normally form the bony calvaria and instead stimulate adjacent underlying cells that can then adopt a cartilage fate. Although we cannot currently distinguish between these models, it is apparent that these decisions are at least partly due to altered Wnt signaling. However, it was difficult to predict how increased FGF ligand expression would affect the overall canonical Wnt signaling pathway since both positive and negative Wnt regulators were upregulated. As such, it was possible that the Wnt pathway might be stimulated, repressed or left unaffected. To distinguish between these possibilities, and to determine whether Wnt dysregulation was contributing to the cranial ossification phenotype, we utilized mice containing *Axin2^lacZ^*. As *Axin2* is an inhibitor of the Wnt pathway, mutations in this gene can increase Wnt signaling compared with controls (Minear et al., 2010). Notably, although homozygous loss of *Axin2* can cause craniosynostosis of the interfrontal/metopic suture, heterozygotes were unaffected (Yu et al., 2005). Therefore, a heterozygous *Axin2^LacZ^* mutation was bred into the *MCAGF8* background to ascertain if there was a genetic interaction between the Wnt and FGF pathways in our model. Significantly, *MCAGF8;Axin2^lacZ^* mice had a less severe phenotype than *MCAGF8* mice with substantial rescue of ossification of the cranial vault; this observation suggests that high levels of *Fgf8b* overexpression cause a downregulation of Wnt signaling. Supporting this hypothesis, previous studies in tissue culture using an osteoblast cell line also demonstrated that FGF signaling was antagonistic to the role of the Wnt pathway in driving bone differentiation (Ambrosetti et al., 2008). Although ossification was partially rescued by the presence of the *Axin2^lac^^Z^* allele, additional patterning defects observed in *MCAGF8* mice were not, including a shortened snout, domed skull and missing eye, indicating that these aspects of *Fgf8b* function are not as responsive to, or do not rely on, Wnt signaling as an intermediate. These latter conclusions were also supported by studies in which we bred the *Axin2^la^^cZ^* mutation into the *MR26F8* background. At the level of gross morphology, the *MR26F8* and *MR26F8;* *Axin2^lacZ^* mice had equivalent craniofacial defects, including coronal suture synostosis, ectopic bone within the orbit, and a misaligned snout accompanied by frontonasal suture defects. Therefore, these pathologies are not rescued in the *MR26F8* model by the presence of the *Axin2^lacZ^* allele, despite the lower levels of *Fgf8b* expression compared with the *MCAGF8* model (L.S. and T.W., unpublished).

The effect of *Fgf8b* overexpression on ossification of bones other than the calvaria was examined using *OC-Cre* transgenic mice to target all developing bones. This approach enabled any differential effect of *Fgf8b* overexpression on intramembranous versus endochondral ossification to be analyzed. In the appendicular and axial skeleton, *OR26F8* and *OCAGF8* mice had a moderate effect on the overall process of osteogenesis, although in common with the *Msx2-Cre Fgf8b* strains there was an effect on limb outgrowth and patterning. These defects, including polydactyly and forelimb-hindlimb fusions, were similar to those previously observed with other models that overexpress the FGF ligands FGF2, FGF4 and FGF8 in the limb (Coffin et al., 1995; Lin et al., 2013; Lu et al., 2006). In the craniofacial skeleton, using either Cre transgene caused a transformation from intramembranous forming skull bone to abnormal unstained matrix, indicating that this transformation can occur regardless of whether *Fgf8b* is expressed from an earlier time point in the ectoderm or from later in the bone progenitors. Interestingly, both transgenes also had similar differential effects on particular bones of the skull vault, with the parietal bones less affected than the frontal bones. In this respect, previous studies suggest that osteoblast location within the skull can affect the degree of FGF signaling activation. Specifically, osteoblasts from bones derived from neural crest cells, such as the frontal bone, express FGF osteogenic ligands and their receptors at higher levels than osteoblasts from bones derived from paraxial mesoderm, such as the parietal (Behr et al., 2010; Quarto et al., 2009). Alternatively, as transgenic *Fgf9* expression has the greatest effect on parietal bone ossification it is possible that these findings reflect the differential activity of particular FGF ligands on skull development (Govindarajan and Overbeek, 2006). Thus, overexpression of *Fgf8b* appears to be more effective at altering cell fate in bones derived from neural crest cells than in those derived from paraxial mesoderm. Future gene expression studies on individual sutures and their associated progenitors might help to further elucidate why particular bones and sutures respond differentially to alteration of specific signaling pathways to cause the various types of craniosynostosis (Brinkley et al., 2016; Teven et al., 2014).

The ability of *OC-Cre* to target other bones in the craniofacial skeleton, such as those of the jaw and cranial base, also extended the observation that the increased *Fgf8b* levels had a greater overall effect on the intramembranous bones, as opposed to those that form through endochondral ossification. One notable exception to the general model of *Fgf8b* action was the supraoccipital bone, which forms through endochondral ossification. Furthermore, this bone showed a differential ossification response depending on whether *Fgf8b* was activated by *Msx2-Cre* versus *OC-Cre*. Thus, although this endochondral bone was not affected in the *OC-Cre* mutants, it was severely hypoplastic in the *MCAGF8* mice. We speculate that the ossification defect in *MCAGF8* mice is caused by an early decrease in *Mef2c* expression, which we detected using RNAseq analysis (Fig. 7D, Table S1), as previous studies have shown that the supraoccipital is particularly sensitive to loss of this gene (Arnold et al., 2007).

Currently, the molecular mechanisms responsible for the early transition from progenitor cell to chondrocyte or pre-osteoblast are not well understood. The availability of these new alleles will allow for further analysis of how different *Fgf8b* expression levels can affect cell-fate decisions during skeletogenesis. Furthermore, alongside other mouse models that manipulate FGF ligands and receptors, these models might help tease apart the combinatorial manner by which specific ligand-receptor interactions can lead to the different forms of craniosynostosis, with potential therapeutic applications. These alleles can also be used to assess how other tissues, such as the dura mater, can influence skull ossification and development (Senarath-Yapa et al., 2012). Finally, our results on the dose-dependent effects of *Fgf8b* on the cranial vault have implications for disease treatment. Some craniosynostosis patients require repeat surgeries owing to the re-fusion of the sutures and a detailed understanding of the molecular pathways downstream of FGF signaling could lead to the rational design of treatments to prevent re-fusion. Unraveling the role of FGF signaling in cranial ossification and its downstream molecular consequences greatly expands our understanding of human craniofacial disorders and provides the possibility of novel treatments for those pathologies.

## MATERIALS AND METHODS

### Mice

#### Mouse strains

All mouse experiments were performed with the approval of the Institutional Animal Care and Use Committee of the University of Colorado Denver. E0.5 was considered to be noon on the day a copulatory plug was found.

We generated two new ROSA26-based Lox-Stop-Lox alleles that can be used to regulate the levels of *Fgf8* expression under the control of Cre recombinase transgene activity: *R26F8* and *CAGF8*. *R26F8* GOF mice have an allele with the endogenous ROSA26 promoter separated from *Fgf8* by a LoxP Stop LoxP cassette. When the Stop cassette is removed by a Cre recombinase, mice express moderate levels of *Fgf8* expression in a tissue-specific manner. Similarly, *CAGF8* GOF mice have an allele with the strong CAG promoter separated from *Fgf8* by a LoxP Stop LoxP cassette. Given the stronger promoter activity of CAG (Qin et al., 2010), removal of the Stop cassette, mediated by Cre recombinase, leads to mice expressing high levels of *Fgf8*.

For *R26^LSL Fgf8b^*, an ∼0.8 kb mouse *Fgf8b* cDNA was PCR-amplified using elongase (Thermo Fisher Scientific Waltham, MA) from a plasmid vector provided by Dr Mark Lewandoski (NCI) using the forward primer Fgf8b FWD and the reverse primer Fgf8b REV (all primer sequences are provided in Table S3). This procedure introduced an *Xho*I site just before an ATG start codon and a *Hin*dIII site downstream of the stop codon. Following subcloning into TA vector (Thermo Fisher Scientific) and sequence confirmation, the insert was digested with *Hin*dIII and this site blunted with the Klenow fragment of DNA polymerase I in the presence of all four dNTPs (all enzymes for cloning were obtained from New England Biolabs, Ipswich, MA). Subsequently, the insert was released using *Xho*I digestion and cloned into the *Xho*I and *Sma*I digested pBTG vector (pBigT-IRES-GFP; Addgene, Cambridge, MA). Next, this insert was released with *Pac*I and *Asc*I and subcloned into the vector pROSA26PAm1 (Addgene) using the same enzymes, to generate the *R26F8* GOF targeting vector.

For *R26^LSL CAG Fgf8b^*, an ∼1.7 kb *Sal*I-*Eco*RI fragment containing the CAG promoter sequence was isolated from the plasmid CAG-GFP (Addgene) and cloned into a vector containing a *Pac*I site upstream of the *Sal*I site as well as *Not*I and *Nhe*I sites downstream of the *Eco*RI site. Subsequently, the *Not*I-*Nhe*I restriction fragment from PGKneotpAlox2 (Addgene) which contains the floxed selection cassette was cloned downstream of the CAG promoter. Finally, the new *Pac*I-*Nhe*I insert fragment was inserted into the previous *Fgf8* GOF targeting vector to generate the *CAGF8* GOF targeting vector. Both targeting vectors were then linearized with *Mlu*I, gel-purified and then electroporated into 129S1/Sv W9.5 embryonic stem (ES) cells.

*R26^LSL Fgf8b^* ES cell clones were screened using the primer pairs (RF5+BTR) and (GFP F+R3R) to detect appropriate homologous recombination with the endogenous *R26R* locus at the 5′ and 3′ end, respectively. PCR reactions employed KOD Hot Start DNA polymerase, as recommended by the manufacturer (EMD Millipore, Billerica, MA). Only clones that were targeted correctly produced a 1.35 kb band (5′ end) and ∼4.8 kb band (3′ end) and these were karyotyped before injection into blastocysts. *R26^LSL CAG Fgf8b^* ES clones were screened in analogous fashion using the primer pairs (RF5+CMV R1) and (GFP F+R3R) and produced similar sized bands at both the 5′ and 3′ ends following homologous recombination.

Following germline transmission, both the *R26F8* and *CAGF8* mice were maintained on an outbred Black Swiss background and eventually bred to homozygozity and then maintained as homozygous colonies. *Msx2-Cre* mice [*(Msx2-Cre)5Rem*] were supplied and originally described for their use in limb studies by Rob Maxson (Sun et al., 2000). ROSA26 Cre reporter mice [*B6.129S4-Gt(ROSA)26Sor^tm1Sor^/J*], Axin2^lacZ^ (*B6.129P2-Axin2^tm1Wbm^/J*) and *OC-Cre* [*B6N.FVB-Tg(BGLAP-cre)1Clem/J*] were obtained from Jackson Laboratory (Bar Harbor, ME). Two points should be noted: (1) as mice with Cre alleles are being bred to homozygous GOF mice, all offspring will inherit a GOF allele and mutants with an activated GOF allele can be identified by the presence of a Cre allele; and (2) the combination of the Cre alleles and the GOF alleles leads to sirenomelia (*R26^LSL Fgf8b^*) or perinatal lethality (*R26^LSL CAG Fgf8b^*), therefore, it is not possible to generate mice containing these Cre recombinase transgenes with homozygous recombined GOF alleles.

#### Genotyping

PCR-based genotyping was performed using DNA extracted from yolk sacs or tail clips using DirectPCR Lysis Reagent (Viagen Biotech, Los Angeles, CA) plus 10 μg/ml proteinase K (Roche, Basel, Switzerland) followed by heat inactivation at 85°C for 45 min. Mutants were identified by PCR using the Qiagen DNA polymerase kit, including the optional Q Buffer solution (Qiagen, Valencia, CA). Mice carrying *Msx2-Cre* or *OC-Cre* transgenes were identified using the primer pair Cre1 and Cre3 at an annealing temperature of 70°C, yielding a band at ∼450 bp. *R26R* mice and the *Axin2^lacZ^* allele were identified using the primer pairs oIMR0039 and oIMR0040. The *R26^LSL Fgf8b^* allele was identified using the primer pair ROSA F and BTR, whereas the primer pair ROSA F and CMV R1 was used to identify the *R26^LSL CAG Fgf8b^* allele. In both cases, ROSA F+ROSA R was used to identify for the presence of the wild-type allele.

### Skeletal analysis

#### Bone and cartilage staining

Embryos, pups and adult mice were collected at appropriate time points and processed as previously described (McLeod, 1980). In brief, following euthanasia and the removal of the skin and organs, the mice were dehydrated in ethanol for a minimum of 3 days before being incubated in acetone for at least 2 days. Subsequently, they were incubated in staining solution comprising ethanol (70%), acetic acid (5%), Alcian Blue (0.3%) and Alizarin Red (0.1%) at 37°C for a minimum of 5 days before being cleared in 2% KOH. In skeletal preparations where Toluidine Blue was utilized, 0.1% Toluidine Blue was added to the above staining solution for 1 h before clearing.

#### Cartilage staining

Embryos (E13.5-E16.5) were collected and processed as previously described (Jegalian and De Robertis, 1992). In brief, embryos were fixed in Bouin's solution for 2 h followed by a series of washes in a solution of 70% ethanol plus 0.1% NH_4_OH until there was no remaining yellow (Bouin's) color. Next, the embryos were equilibrated in 5% acetic acid (2×1 h washes) and incubated overnight in a solution of Alcian Blue (0.05%) and acetic acid (5%). The embryos were then washed twice with 5% acetic acid (∼1 h washes) and twice with methanol (minimum 1 h washes). Finally, the embryos were cleared in BABB (1:2 benzyl alcohol/benzylbenzoate).

#### Section histochemistry

For sections from E18.5 embryos, as well as P0 and P12 pups, the embryos/pups were fixed in 70% ethanol (skin removed from P12 pups first). They were then sent to the Yale Orthopedic Histology and Histomorphometry Laboratory for plastic embedding and sectioning, as well as staining. Four stains were utilized: Goldner's trichome (Gruber, 1992), von Kossa (George Clark, 1981), Alcian Blue+PAS (Mowry, 1956) and Toluidine Blue (Sridharan and Shankar, 2012). Suture widths were calculated as the average of six sections: two sections each from three biological replicates.

For E16.5 sections, the embryos were fixed overnight in 4% paraformaldehyde (PFA). Following fixation, tissue was dehydrated in a graded series of ethanol and xylene and subsequently embedded in paraffin. After embedding, the embryos were sectioned with a Leica RM 2235 at 10 µm onto charged glass slides. After drying, sections were deparaffinized through a graded series of xylene and ethanol to 70% ethanol. The sections were then stained in 0.04% Toluidine Blue O in 0.1 M sodium acetate buffer (pH 4.0), rinsed briefly and counterstained with methyl green (Vector Laboratories, Burlingame, CA), as per the manufacturer's instructions. Coverslips were applied with Fluoromount-G (Southern Biotech, 0100-01).

### *In situ* hybridization/immunofluorescence

Probes were generated by cloning a unique fragment (primer sequences given upon request) into a TOPO vector (Life Technologies, Grand Island, NY), using cDNA synthesized from mouse embryonic mRNA as a template. cDNA was generated using the Superscript^®^ III First-Strand Synthesis System (Life Technologies), as per the manufacturer's instructions. Sequence-verified plasmids were linearized and antisense probes synthesized using an appropriate DNA-dependent RNA polymerase (T7/T3/SP6) and DIG RNA labeling mix (Roche).

Embryos were fixed in 4% PFA overnight at 4°C and then incubated in 30% sucrose in phosphate-buffered saline (PBS) at 4°C until the embryos sank to the bottom of the well (overnight to several days). The embryos were then incubated in a 1:1 solution of 30% sucrose: OCT (Sakura Finetek, Torrence, CA), rocking at 4°C, overnight for several days. Finally, the embryos were transferred to 100% OCT and rocked at 4°C for at least 30 min before being embedded in OCT on dry ice and stored at –80°C until sectioning. Sections were cut at 10 μm on a Leica CM cryostat (Leica Biosystems, Buffalo Grove, IL) and mounted on aminopropyltriethoxysilane (APES)-coated slides. APES-coated slides led to better retention of sections during processing than traditional charged glass slides and were made as previously described (www.methodbook.net/probes/insitu.html).

Subsequently, sections were prehybridized in slide mailers following five steps: (1) fixed for 10 min in 4% PFA/PBS at room temperature, followed by three washes in PBS for 3 min each; (2) digested in proteinase K (1 µg/ml in 50 mM Tris pH 7.5, 5 mM EDTA) for 4 (E12.5), 6 (E14.5) or 8 (E16.5) min; (3) refixed in 4% PFA/PBS for 5 min at room temperature, followed by three washes in PBS for 3 min each; (4) acetylated (1.36% triethanolamine, 0.178% HCL, 0.2544% acetic anhydride in water) for 10 min at room temperature, followed by three washes in PBS for 5 min each; (5) incubated in hybridization buffer [50% formamide, 5× saline sodium citrate (SSC) pH 4.5, 50 µg/ml yeast tRNA, 1% SDS, 50 µg/ml heparin] at 55°C for 1-2 h. Next, sections were hybridized as follows: (1) incubated in hybridization buffer plus 1 ng/µl probe overnight at 70°C; (2) submerged in prewarmed (70°C) 5×SSC pH 7 and incubated on rocker for 30 min at room temperature; (3) incubated in prewarmed (70°C) 0.2×SSC (pH 7) for 3 h at 70°C, followed by incubation in fresh 0.2×SSC for 5 min at room temperature; (4) incubated in 1× maleic acid buffer (MAB, pH 7.5) for 5 min at room temperature; (5) incubated in blocking solution [2% blocking reagent (Roche, 11096176001), 10% heat-inactivated sheep serum, 0.1% Tween-20, all in 1×MAB] for 1 h at room temperature, followed by incubation in blocking solution plus anti-DIG antibody (1:5000, FAB fragments, Roche) overnight at 4°C. Finally, sections were washed and stained following five steps, all at room temperature: (1) washed three times in 1×MAB with 0.1% Tween-20 for 15-30 min; (2) washed in DEPC-H_2_O with 0.1% Tween-20 for 20 min; (3) slides were removed from mailers and 200 µl of BM Purple (Roche) with 0.1% Tween-20 was added to each slide; (4) slides were kept in dark until desired signal observed; (5) slides were counterstained with nuclear fast red (Vector Laboratories) for 10 min and rinsed with water. Finally, coverslips were applied with Fluoromount-G (Southern Biotech, 0100-01).

### Alkaline phosphatase staining

#### BM Purple

Sections were first washed in PBS at room temperature for 10 min and then washed in DEPC-H_2_O with 0.1% Tween-20 for 20 min. Next, 200 µl of BM Purple (Roche) with 0.1% Tween-20 was added to each slide and the slides were kept in the dark until the desired signal was observed. Finally, slides were counterstained with nuclear Fast Red for 10 min and rinsed with water. Coverslips were applied with Fluoromount-G.

#### Liquid Fast-Red

Sections were first washed in PBS at room temperature for 10 min. Next, 200 µl of liquid Fast-Red solution (Abcam, ab64254) was added to each slide and slides were kept in the dark until the desired signal was observed. Finally, slides were counterstained with methyl green (Vector Laboratories), as per the manufacturer's instructions. Coverslips were applied with Fluoromount-G.

### β-Galactosidase staining

#### Whole mount

β-Galactosidase staining of whole embryos was carried out as follows. Embryos were fixed for 1 h at 4°C in 4% PFA in PBS. Next, they were rinsed three times for 10-30 min at room temperature in lacZ rinse buffer (0.2 M sodium phosphate, pH 7.3; 2 mM magnesium chloride; 0.02% NP40; 0.01% sodium deoxycholate). The embryos were then rocked overnight in the dark at room temperature in lacZ staining solution (lacZ rinse buffer containing 5 mM potassium ferricyanide, 5 mM potassium ferrocyanide and 1 mg/ml X-gal). Finally, the reaction was stopped by transferring the embryos to PBS and fixing in 4% PFA.

#### Sections

β-Galactosidase staining of frozen sections was carried out as described previously (Chai et al., 2000). In brief, embryos were fixed in 0.2% glutaraldehyde for 30 min at room temperature. They were then soaked at 4°C in 10% sucrose in PBS for 30 min, followed by PBS plus 2 mM MgCl_2_, 30% sucrose and 50% OCT for 2 h at 4°C. Next, the embryos were embedded in 100% OCT on dry ice. Sections were cut at 10 μm and mounted on charged glass slides, after which the slides were fixed in 0.2% glutaraldehyde for 10 min on ice, rinsed briefly with 2 mM MgCl_2_ in PBS, and washed in 2 mM MgCl_2_ in PBS for 10 min, again on ice. Finally, the sections were incubated in detergent rinse solution (0.005% NP40, 0.01% sodium deoxycholate in PBS) for 10 min at 4°C and stained in X-Gal staining solution (detergent rinse solution plus 1 mg/ml X-Gal; Invitrogen/Life Technologies, Carlsbad, CA) for 2-3 days in the dark at room temperature. Following staining, sections were counterstained with nuclear Fast Red for 10 min and rinsed with water. Finally, coverslips were applied with Fluoromount-G.

### RNA quantification

#### qPCR

Three embryos were collected from each of the four groups: (1) *MR26F8* mutants, (2) *MR26F8* littermate controls, (3) *MCAGF8* mutants and (4) *MCAGF8* littermate controls (12 embryos in total). Skin samples were taken from the region between the eyes and the ears of E18.5 embryos. This region correlates with the region where *Msx2-Cre* was expressed (Fig. S2C) and, thus, the levels of *Fgf8* should vary between the controls and mutants. RNA was extracted from the skin using the RNeasy Fibrous Tissue Mini Kit (Qiagen, 74704), following the manufacturer's instructions, including the DNAse digestion step. Additionally, to ensure DNA removal, the extracted RNA was treated using the TURBO DNA-free Kit (Thermo Fisher Scientific, AM1907). For each sample, 500 ng of RNA was reverse-transcribed to complementary DNA (cDNA) using SuperScript III First-Strand synthesis kit (Invitrogen, 18080051). Real-time PCR reactions were performed using SYBR Select Master Mix (Applied Biosystems, 4472908; Austin, TX) on a CFX Connect Real-Time System (Bio-Rad, Hercules, CA). The expression of *Fgf8* was normalized to that of corresponding *Actb* (β-actin), a housekeeping gene; primers utilized are found in Table S3. Controls (*Msx2-Cre* negative embryos) from *MR26F8* and *MCAGF8* litters had similar *Fgf8* expression levels that were not found to be statistically significantly different (*P*=0.678), thus all six littermate controls were pooled and expression normalized to one. The expression of *Fgf8* in the *MR26F8* and *MCAGF8* mutants was normalized to the combined control sample and presented as fold expression change. Error bars show standard deviation of the fold change.

#### RNAseq

For the bone and cartilage RNAseq, E14.5 embryos, generated from an *Msx2-Cre*×*CAGF8* cross, were dissected in ice-cold PBS. From the mutant and control embryos, the skin was peeled away from the skull and the underlying cranial vault tissue was collected. Subsequently, for the control embryos, the brain was removed and the cartilaginous sections of the cranial base were carefully collected as well. All tissues from the mutant (cranial vault) and control embryos (cranial vault and cartilaginous cranial base) were stored in RNAlater (Ambion/Life Technologies) until RNA extraction. RNA was extracted from nine mutant and control cranial vaults as well as nine cranial bases using RNeasy Lipid Tissue Mini Kit (Qiagen, 74804) following the manufacturer's protocol. Within each group, the nine samples were pooled into three groups, with three samples making up each group, so that the RNA concentrations were similar. Samples were submitted to the University of Colorado Denver Genomic and Microarray Core and sequenced using the Illumina HiSeq4000 Platform and single-end reads (1×151). Reads generated were mapped to the mouse genome by gSNAP, expression derived by Cufflinks, and differential expression analyzed by ANOVA in R, as described previously (Bradford et al., 2015). DAVID (Huang et al., 2009a,b), with default parameters, was used for functional annotation clustering of significantly upregulated and downregulated genes. Data are available at Gene Expression Omnibus (GEO) with the accession number GSE112413.

## Supplementary Material

Supplementary information
