# Supplementary material for: Increased FGF8 signaling promotes chondrogenic rather than osteogenic development in the embryonic skull
Source: Dis Model Mech. 2018 Jun 15;11(6):dmm031526. doi: 10.1242/dmm.031526 (PMC6031357; doi:10.1242/dmm.031526)
Supplement: Supplementary information [file dmm-11-031526-s1.pdf]

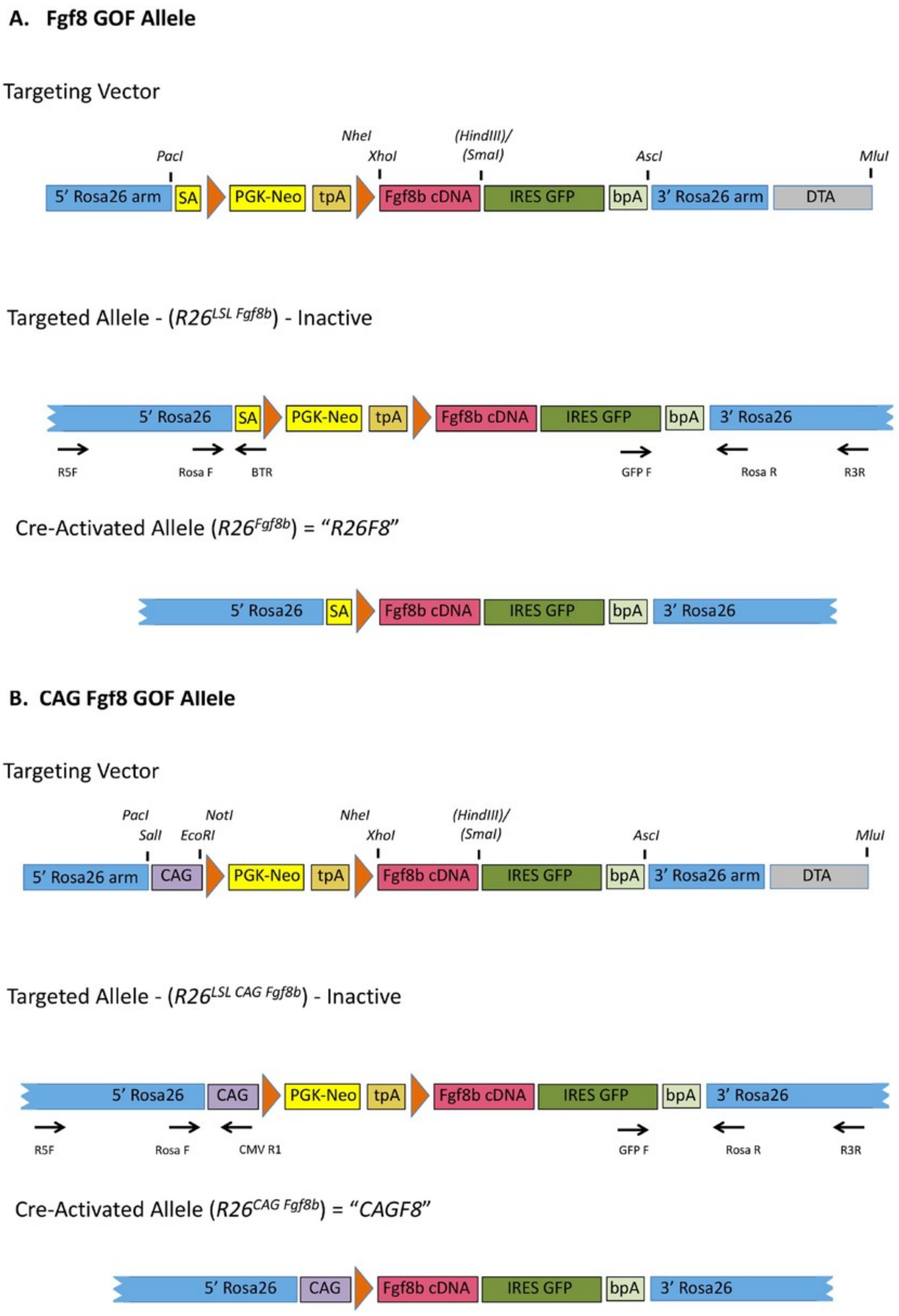

Supplementary Figure 1. Detailed Map of *R26F8* and *CAGF8* constructs.

Disease Models & Mechanisms • Supplementary information

A: Schematic of the *R26F8* allele

Top. The targeting vector showing the standard homology arms used for targeting the *Gt(ROSA)26Sor* locus (*ROSA26*). The *ROSA26* locus provides a promoter and a non-coding first exon to generate mRNA transcripts. The splice acceptor site (SA), *LoxP* sites (orange triangles), the drug selection cassette (PGK-Neo), and multiple poly A addition sequences to prevent transcription read-through (tpA), along with the IRES-GFP and the bovine growth hormone poly A addition sequences (bpA) are derived from the pBTG vector (see Materials and Methods). DTA indicates the diphtheria toxin A negative selection cassette. The sites of restriction enzymes used for cloning and linearization are shown, but note that these are not necessarily unique sites within vector sequences.

Middle. The allele after homologous recombination with the sites of primers used for ES cell screening and genotyping. Note that the primers R5F and R3R used to screen for appropriate homologous recombination lie outside the homology arms. For standard mouse genotyping ROSA F + ROSA R give an ~165bp wildtype band. ROSA F + BTR give an ~250bp band for the targeted allele.

Bottom. The allele after Cre mediated recombination that allows generation of a functional *Fgf8b* transcript. Note, this allele is abbreviated as *R26F8* in the text

Also note that in all instances the various DNA elements are not shown to scale.

B: Schematic of the *CAGF8* allele

Top. The targeting vector as in A. except that a CAG enhancer/promoter element has been inserted upstream of the positive selection cassette. The sites of restriction enzymes used for cloning and linearization are shown, but note that these are not necessarily unique sites within vector sequences.

Middle. The allele after homologous recombination with the sites of primers used for ES cell screening and genotyping. Note that the primers R5F and R3R used to screen for appropriate homologous recombination lie outside the homology arms. For standard mouse genotyping ROSA F + ROSA R give an ~165bp wildtype band. ROSA F + CMV R1 give an ~230bp band for the targeted allele.

Bottom. The allele after Cre mediated recombination that allows generation of a functional *Fgf8b* transcript. Note, this allele is abbreviated as *CAGF8* in the text

Also note that in all instances the various DNA elements are not shown to scale.

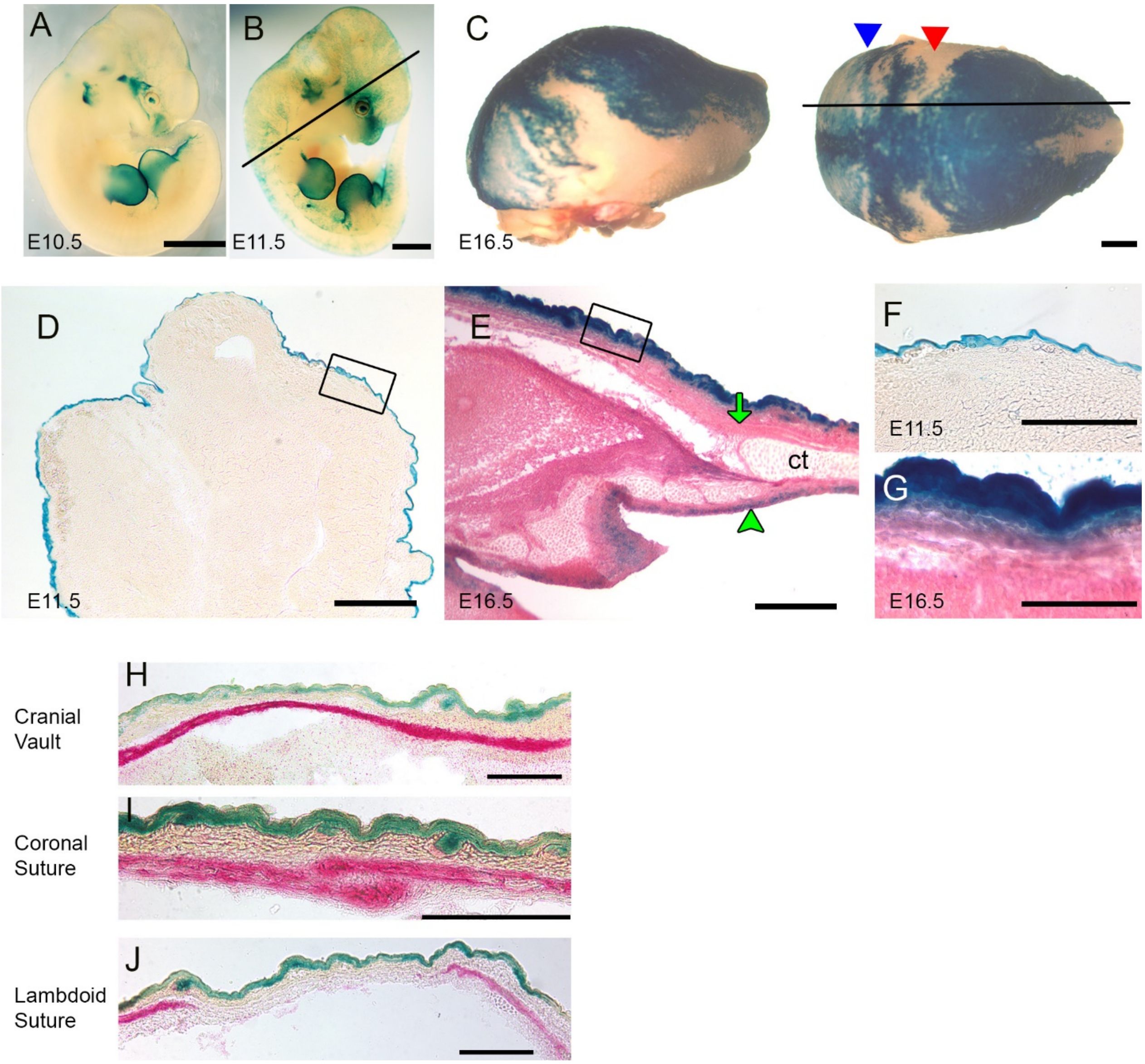

Supplemental Figure 2. Embryonic expression of *Msx2-Cre* during craniofacial development.

Msx2-Cre mediated recombination was visualized using β-galactosidase staining (blue/green) of *ROSA26 LacZ Reporter* embryos. (A-C) Lateral views of whole mount β-galactosidase staining on E10.5 (A) and E11.5 (B) embryos. (C) At E16.5, almost the entire dorsal aspect of the head showed β-galactosidase staining (lateral view on the left, dorsal view on the right). Red and blue triangles denote regions over part of the parietal and intraparietal bones, respectively, where *Msx2-Cre* mediated recombination did not occur as efficiently. Black lines in B and C indicate plane of section in D, F and E, G respectively. (D-G) β-galactosidase staining of frozen sections at E11.5 in a frontal plane (D, F) and E16.5 in a sagittal plane (E, G), counterstained with nuclear fast red. F and G are magnifications of the regions outlined by black boxes in D and E,

Disease Models & Mechanisms • Supplementary information

respectively. Sections are shown at both 10x (D, E) and 40x (F, G) magnification. Note in E, bone (arrow) and cartilage (ct), do not show β-galactosidase staining, whereas staining occurs in both ectoderm (boxed) and nasal epithelium (arrowhead). (H-J): β-galactosidase staining of frozen sagittal sections of the cranial vault of E16.5 *ROSA26 LacZ Reporter* embryos showing (H): the anterior cranial vault, (I): the coronal suture, and (J): the lambdoid suture. Sections were stained with liquid fast red to highlight bone (magenta). Note that β-galactosidase staining is found only in the ectoderm, not the cranial bone or sutures. Scale bars: A-C: 500μM; D-E: 100μM; F-G: 50μM; H-J: 200 μM.

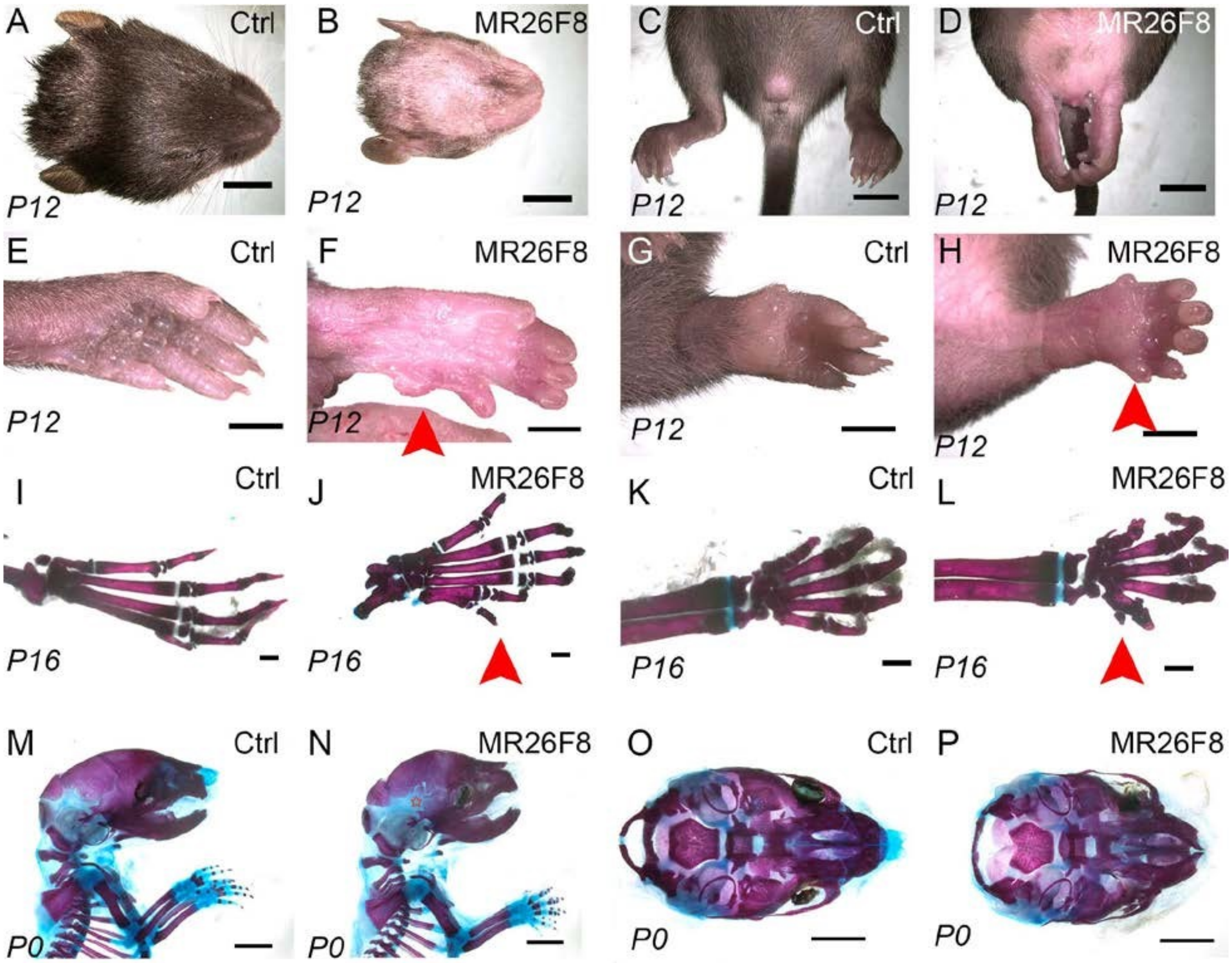

Supplementary Figure 3. ***MR26F8*** **Cranial and Limb Phenotypes**

(A-H): Gross morphological view of P12 heads (A-B) and hindlimbs (C-D) of the control (A, C, E, G) and *MR26F8s* (B, D, F, H). (I-L): Bone and cartilage staining of P16 limbs of controls (I, K) and *MR26F8s* (J, L). (E-F, I-J): hindlimb; (G-H, K-L): Forelimb. Red arrowheads denote extra digits on the posterior side of the *MR26F8* limb (postaxial polydactyly). (M-P): Skeletal staining of P0 control (M, O) and *MR26F8* (N, P) mice showing lateral views of skulls and forelimbs (M, N) and ventral view of the cranial base after removal of the mandible (O, P). Red star in N denotes loss of ossification of the parietal. Scale Bars: 1mm.

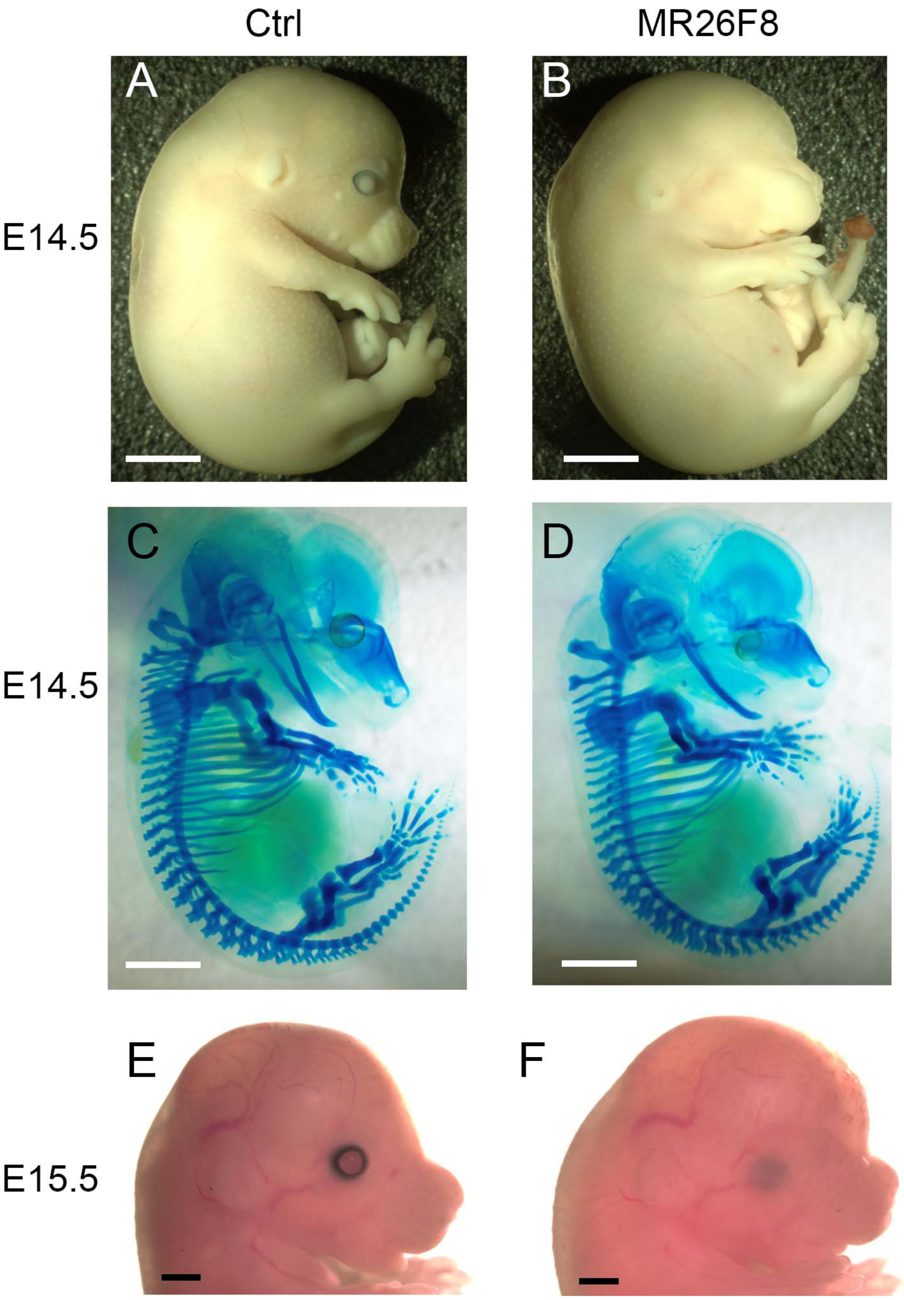

Supplemental Figure 4. ***MR26F8*** **E14.5 and E15.5 phenotypes.**

(A-D): Gross morphology (A-B) and cartilage stain (C-D) of E14.5 control (A, C) and *MR26F8* (B, D) embryos. (E-F): Gross morphology of E15.5 control (E) and *MR26F8* (F) heads. Note embryos in (A-B) have been fixed prior to photography. Scale Bars= A-D, 2mm; E-F, 1mm.

Disease Models & Mechanisms • Supplementary information

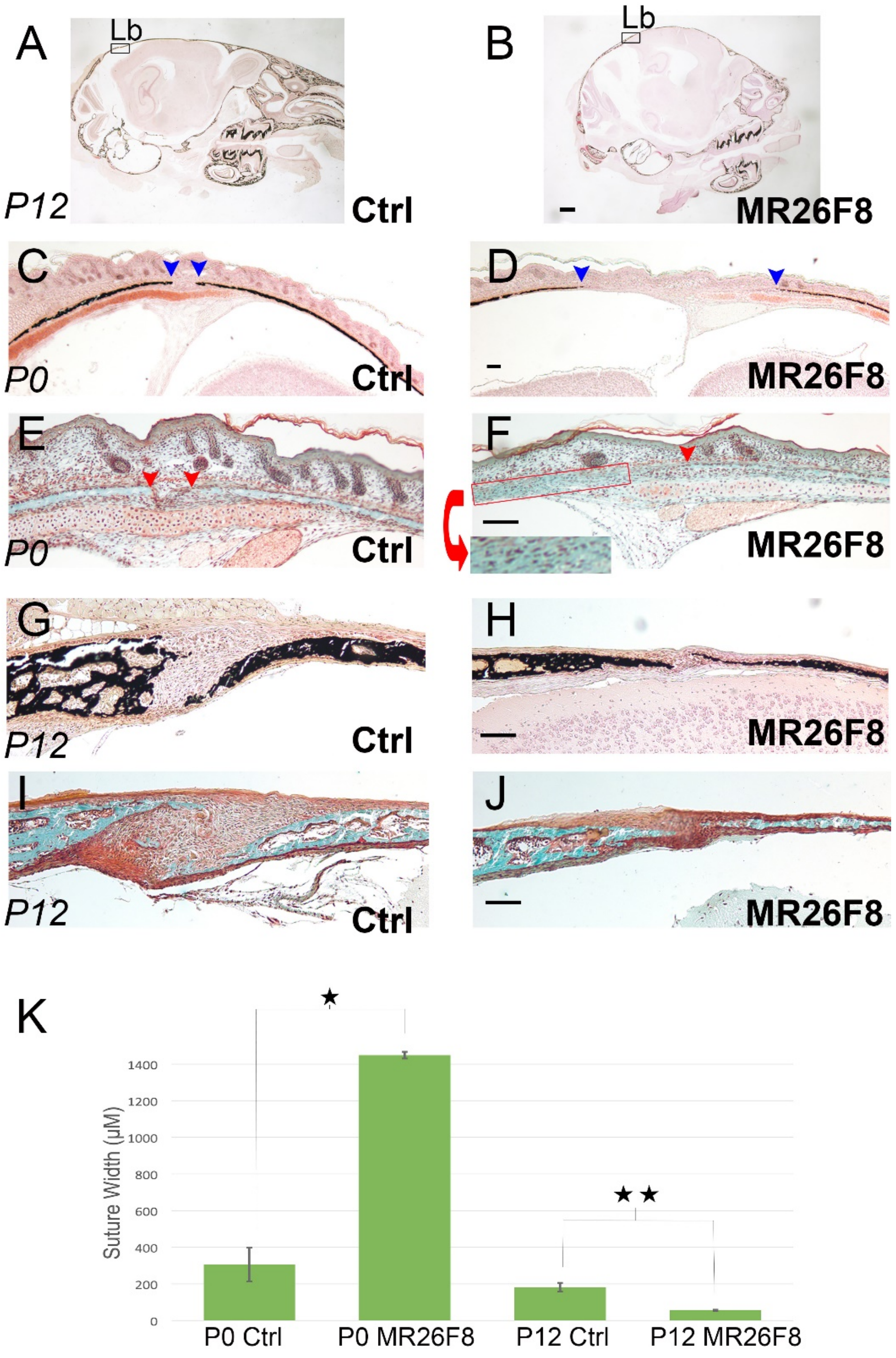

Supplemental Figure 5. ***MR26F8*** **mutants have delayed ossification, followed by over ossification, of the lambdoid suture.**

Disease Models & Mechanisms • Supplementary information

Low magnification images in (A, B) show the approximate position of the lambdoid suture (Lb) from P12 sagittal sections. Sagittal sections from skulls of P0 (C-F) and P12 (G-J) control (C, E, G, I) and *MR26F8* (D, F, H, J) pups. Sections (A-D, G-H) were stained with von Kossa, such that mineralized bone appears dark red/black. Sections (E-F, I-J) were stained with Goldner's Trichome stain such that mature bone matrix appears green whereas immature bone matrix stains red. In the P0 sections, blue arrowheads mark the limits of mineralization (C-D); red arrowheads show the extent of the unmineralized mature osteoid (E-F). In F, only one side of the mature osteoid is shown, with a mixture of mature and immature bone matrix occurring between the mature osteoid in the mutant as shown in greater detail in the inset (red arrow). The bar graph (K) shows the width of the lambdoid sutures at P0 and P12. Comparison of the width between the controls and *MR26F8s* is significantly different at both P0 ($p<0.0001$) and P12 ($p<0.0003$). Bars show average suture width (µM); error bars denote standard error within the group. Scale Bars: A-B: 1mm; C-J: 100µM. Aged-matched controls and mutants are at the same scale.

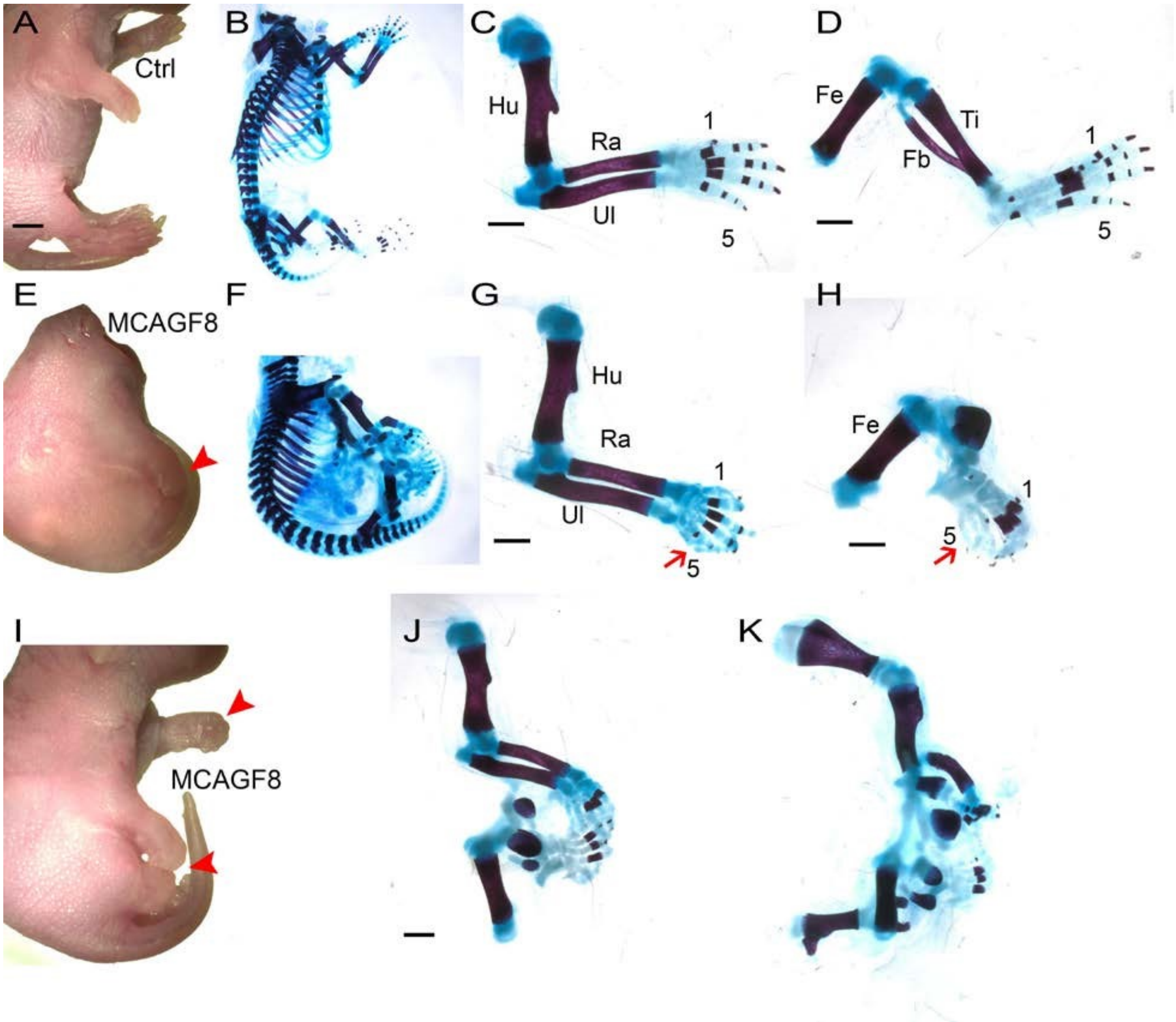

Supplementary Figure 6. ***MCAGF8*** **Limb Phenotypes**

E18.5 gross (A, E, I) and skeletal (B-D, F-H, J-K) limb phenotypes. (A-D): controls, (E-K) *MCAGF8s*. The latter exhibit a wide range of phenotypes from polydactyly in the forelimb (G, compared to control, C) and hindlimb (H, compared to control, D) to fused forelimbs and hindlimbs (less severe, J; more severe, K). Red arrowheads denote various gross limb phenotypes. Red arrows denote postaxial polydactyly with 1 indicating the anterior most digit and 5 indicating the posterior most digit. Abbreviations: Fb, fibula; Fe, femur; Hu, humerus; Ra, radius; Ti, tibia; Ul, ulna. Scale Bars= 1mm.

Disease Models & Mechanisms • Supplementary information

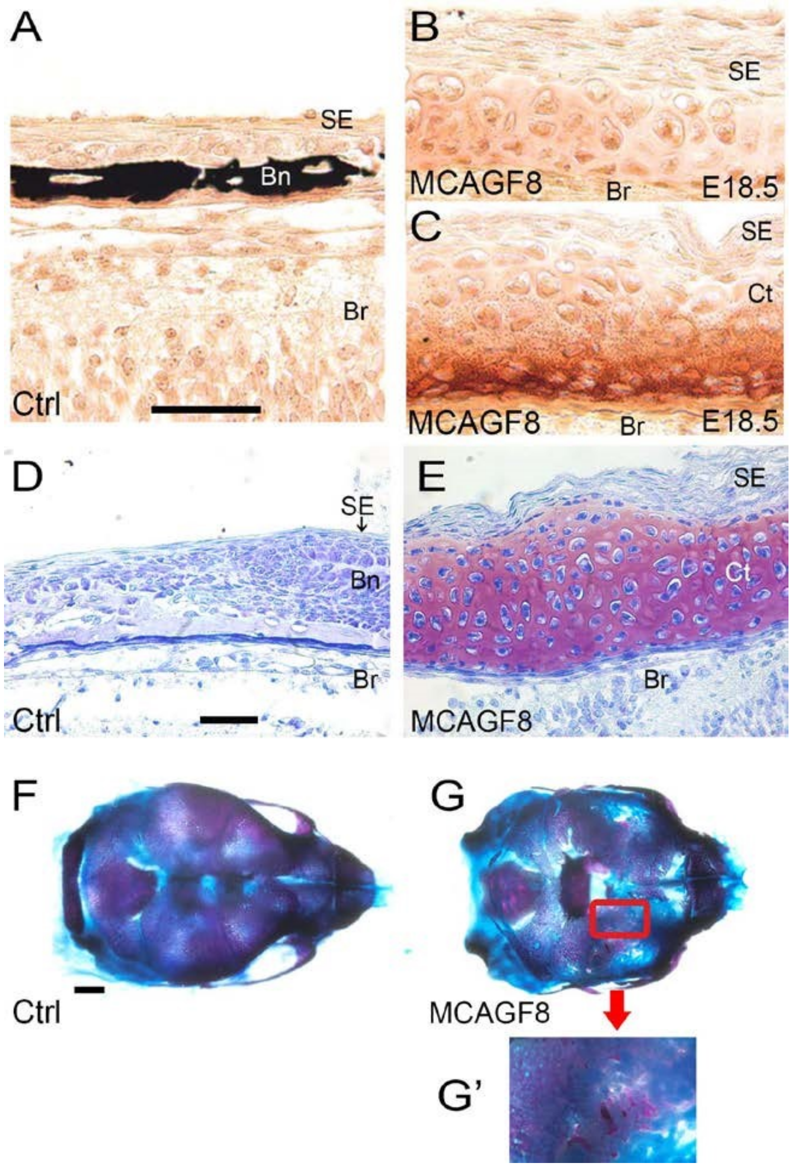

Supplemental Figure 7. Abnormal cartilage replaces intramembranous bone in *MCAGF8* mice.

(A-E) Frontal sections through the parietal bones of the E18.5 controls and equivalent location in *MCAGF8s*; regions shown are midway between the dorsal and ventral most aspects of the parietal. (A, C) von Kossa stain of the control (A) and mutant (B, C) sections. Mineralized bone is stained in dark red/black. (D, E) Toluidine blue staining of control (D) and *MCAGF8* (E) sections. The nuclei stain blue and the glycosaminoglycans that make up the cartilage matrix stain reddish purple. (F-G): Dorsal view of E18.5 skulls after bone and cartilage staining with alizarin red, alcian blue, and toluidine blue (cartilage). The red rectangle (G) outlines a region where cartilage and bone overlap. G' shows that same region magnified. Abbreviations: Bn, bone; Br, brain; Ct, cartilage; SE, surface epithelium. Scale bars: A, D: 20µM, F: 1mm. Aged matched controls and mutants are at the same scale.

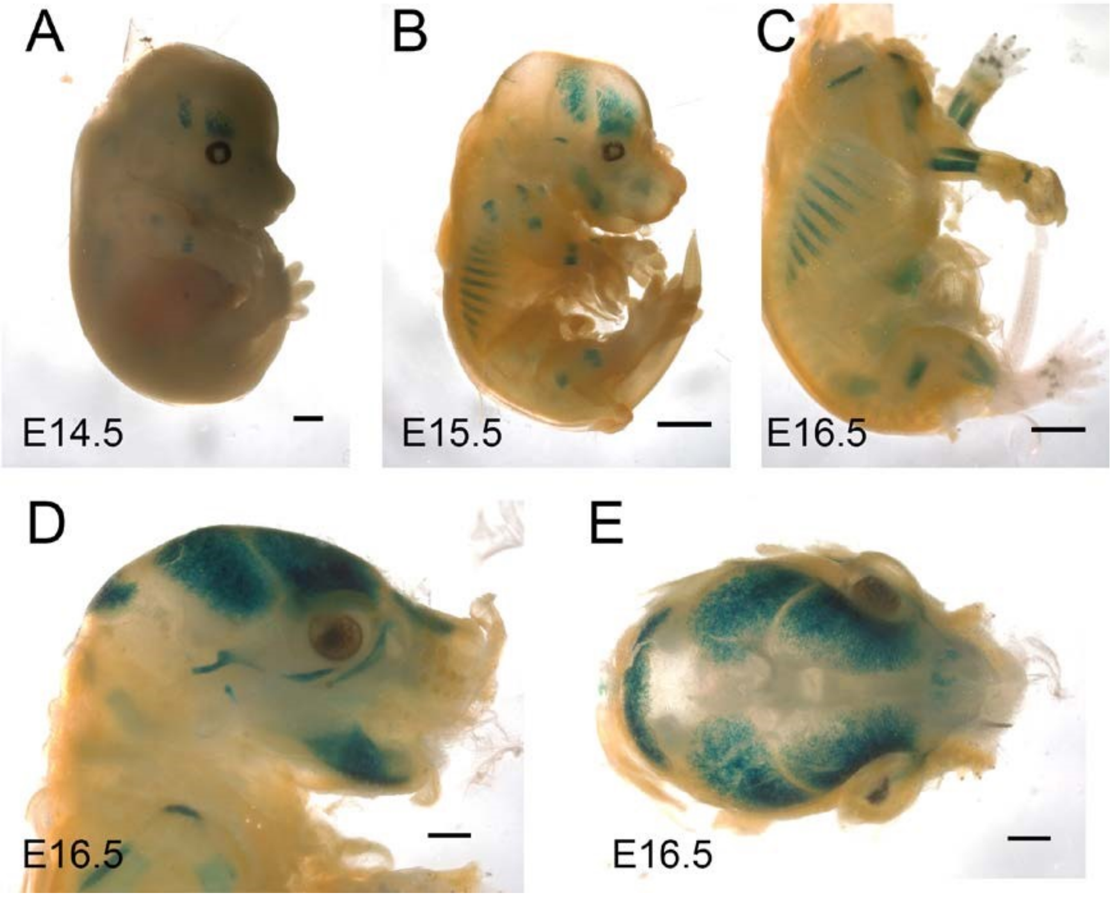

Supplemental Figure 8. *Osteocalcin-Cre* expression from E14.5-E16.5

Osteocalcin-Cre (*OC-Cre*) mediated recombination was visualized using β-galactosidase staining (blue) of *ROSA26 LacZ Reporter* embryos. (A-E) Whole embryo β-galactosidase staining was performed at E14.5 (A), E15.5 (B), and E16.5 (C-E). Skin was removed prior to staining in the E15.5 and E16.5 embryos to allow stain penetration. (C): Lateral view of the ribs and limbs. (D): Lateral view of the head. (E): Dorsal view of the cranial vault. Scale Bars= A, DE: 1mm; B, C: 2mm.

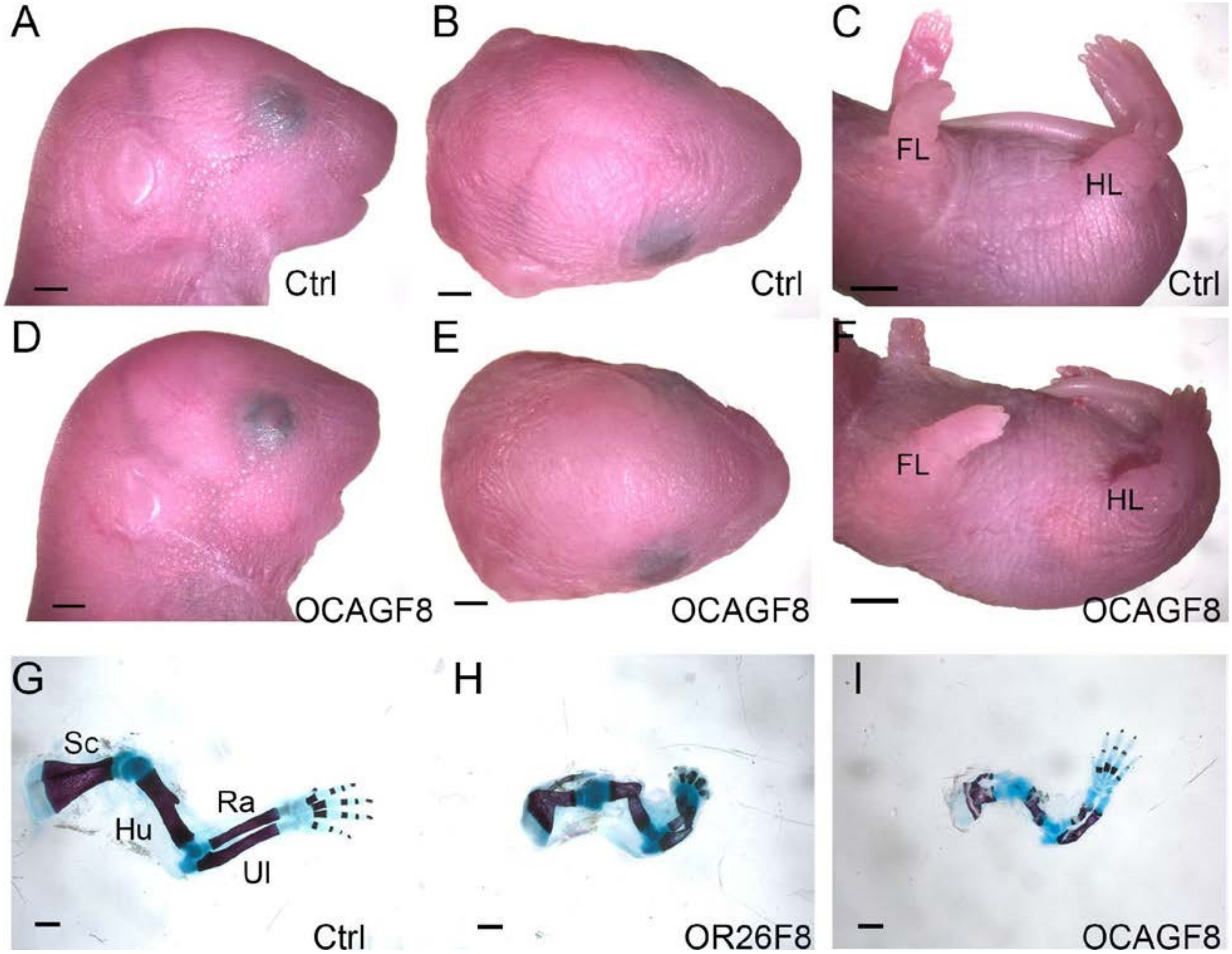

Supplemental Figure 9. Gross morphology and skeletal analysis of forelimbs in E18.5 *OR26F8* and *OCAGF8* embryos

(A-F) Gross morphology of E18.5 control (A-C) and *OCAGF8* (D-F) embryos. (A, D) Lateral view of head. (B, E) Dorsal view of the head. (C, F) Lateral view of the body. (G-I): Bone and cartilage staining of E18.5 forelimbs from control (G), *OR26F8* (H), and *OCAGF8* (I) embryos. Mutants are shown at the same magnification as the controls. Abbreviations: FL, forelimb; HL, hindlimb. Hu, humerus; Ra, radius; Sc, scapula; Ul, ulna. Scale Bars = A-B, D-E, G-I: 1mm; C, F: 2mm.

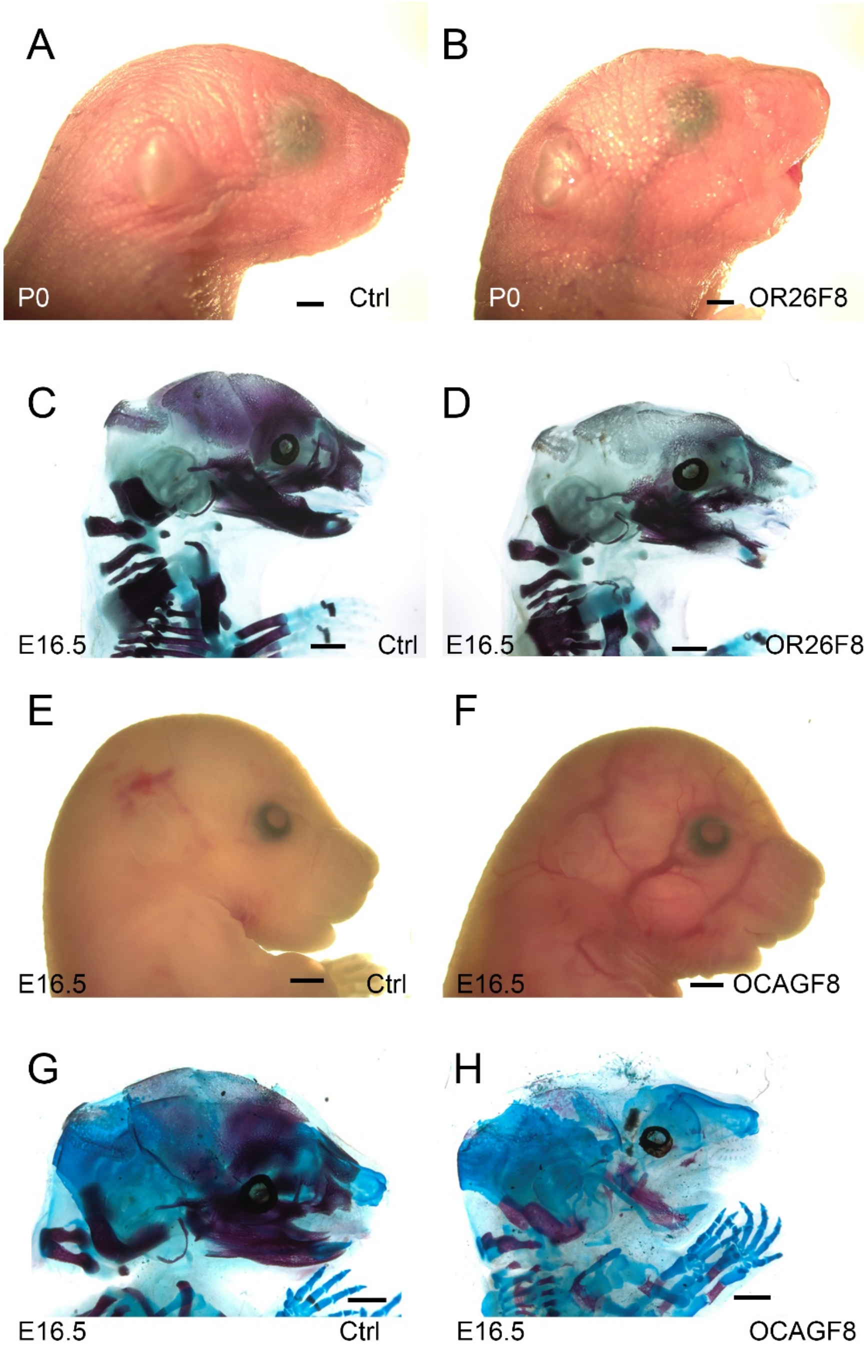

Supplemental Figure 10. *OR26F8* and *OCAGF8* mice have craniofacial and skeletal defects.

(A-B): Gross morphology of P0 control (A) and *OR26F8* (B) heads, lateral view. (C-D): Bone and cartilage staining of E16.5 control (C) and *OR26F8* (D) heads, lateral view. (E-F): Gross morphology of E16.5 control (E) and *OCAGF8* (F) heads, lateral view. (G-H) Bone and cartilage staining of E16.5 control (G) and *OCAGF8* (H) heads, lateral view. Note that for the bone and cartilage staining shown in panels C, D, G, H a reduced concentration of alcian blue was employed so that bone stained with alizarin red could be more readily visualized in the

Disease Models & Mechanisms • Supplementary information

mutants. In addition, the skulls of the mutants were more fragile than the controls and so it was only possible to clear the embryos for a limited time before they were too damaged for photodocumentation. This leads to greater trapping of alcian blue, especially in (H), than would normally occur. Scale bars= 1mm.

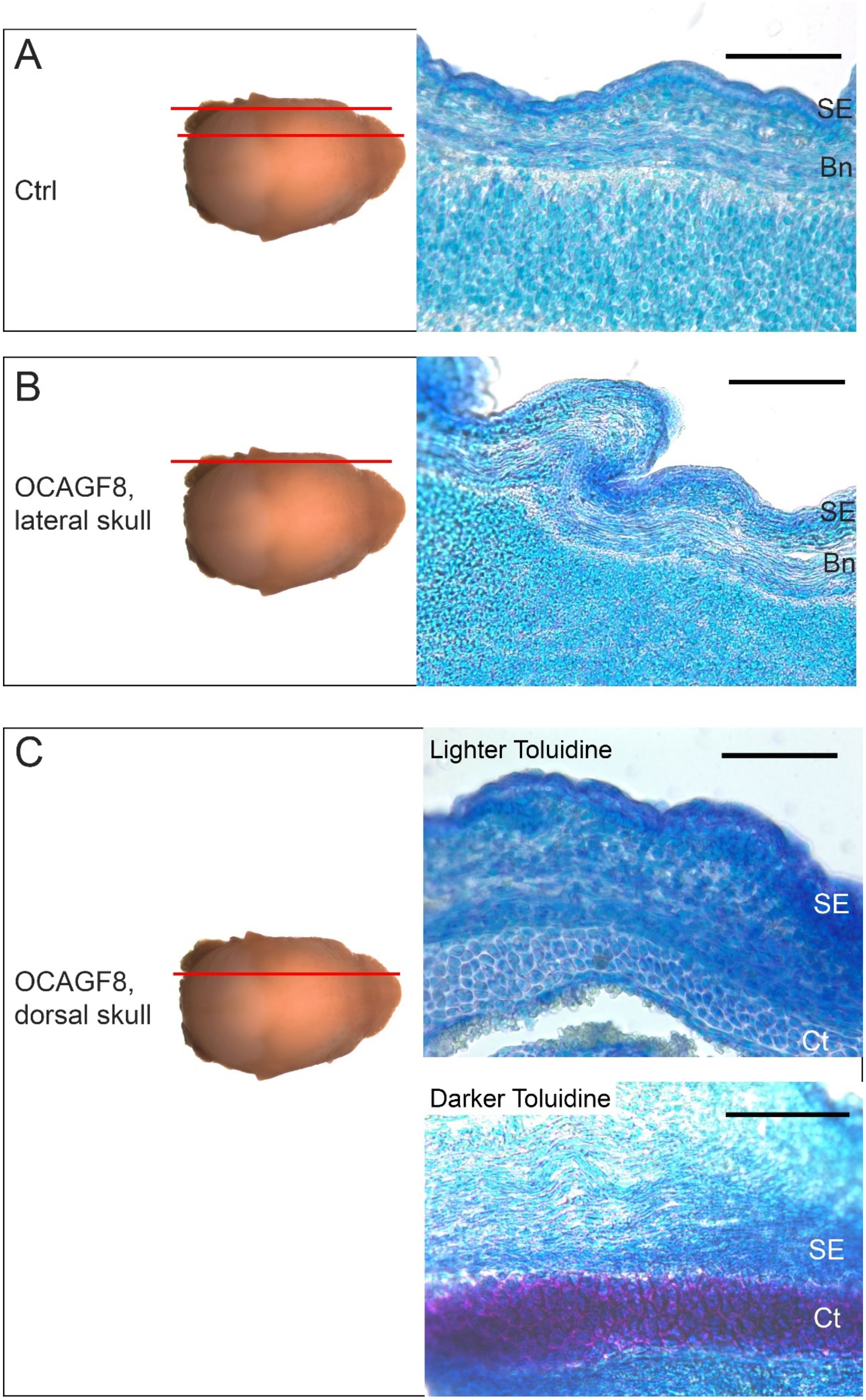

Supplemental Figure 11. Cartilage formation in the *OCAGF8* dorsal skull at E16.5

(A-C): Sagittal sections of E16.5 control (A) or *OCAGF8* (B, C) skulls stained with toluidine blue and methyl green. Red lines superimposed on E16.5 embryo heads show plane of dissection. Note the cartilage in the dorsal (C), but not lateral (B) *OCAGF8* E16.5 skull. In contrast, both the dorsal (A) and lateral skull sections (not shown) were similar in the controls. In C, the cartilage/lacunae morphology can be visualized in the lighter stained cartilage (top), whereas the characteristic reddish purple toluidine cartilage stain can be seen in the darker stained cartilage (bottom). Abbreviations: Ct, cartilage; SE, surface epithelium. Scale Bars = 80 μM.

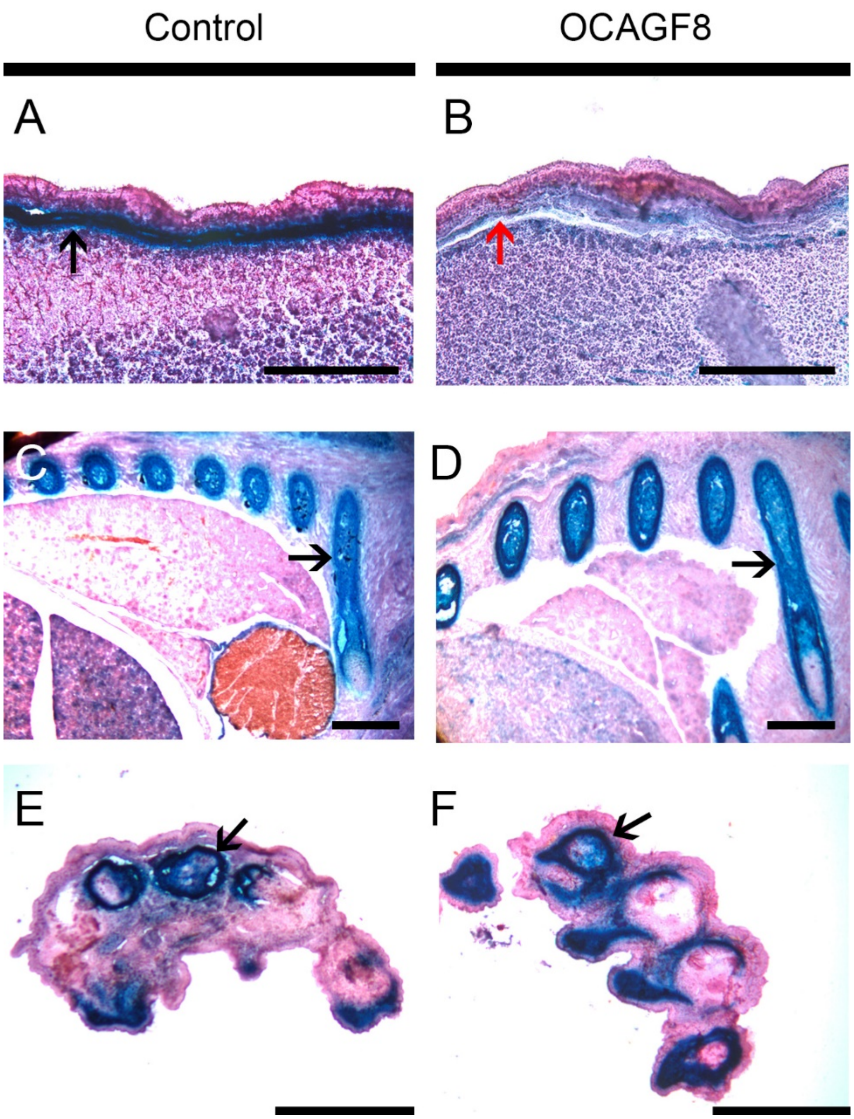

Supplemental Figure 12. Alkaline Phosphatase staining in *OCAGF8* intramembranous and endochondral bones

Sections of E16.5 control (A, C, E) and OCAGF8 (B, D, F) stained with BM Purple to detect alkaline phosphatase activity and counterstained with nuclear Fast Red. (A, B) Sagittal sections of cranial vault; (C, D) sagittal sections of trunk showing ribs; (E, F) frontal sections of forelimb digits. Black arrows indicate regions of alkaline phosphatase activity (blue) in the control cranial vault (intramembranous bones) as well as the ribs and digits (endochondral bones) in both the controls and mutants. Red arrow indicates absence of activity in cranial vault of mutant. Scale Bars = A-B, 200 μM; C-F, 500 μM.

Disease Models & Mechanisms • Supplementary information

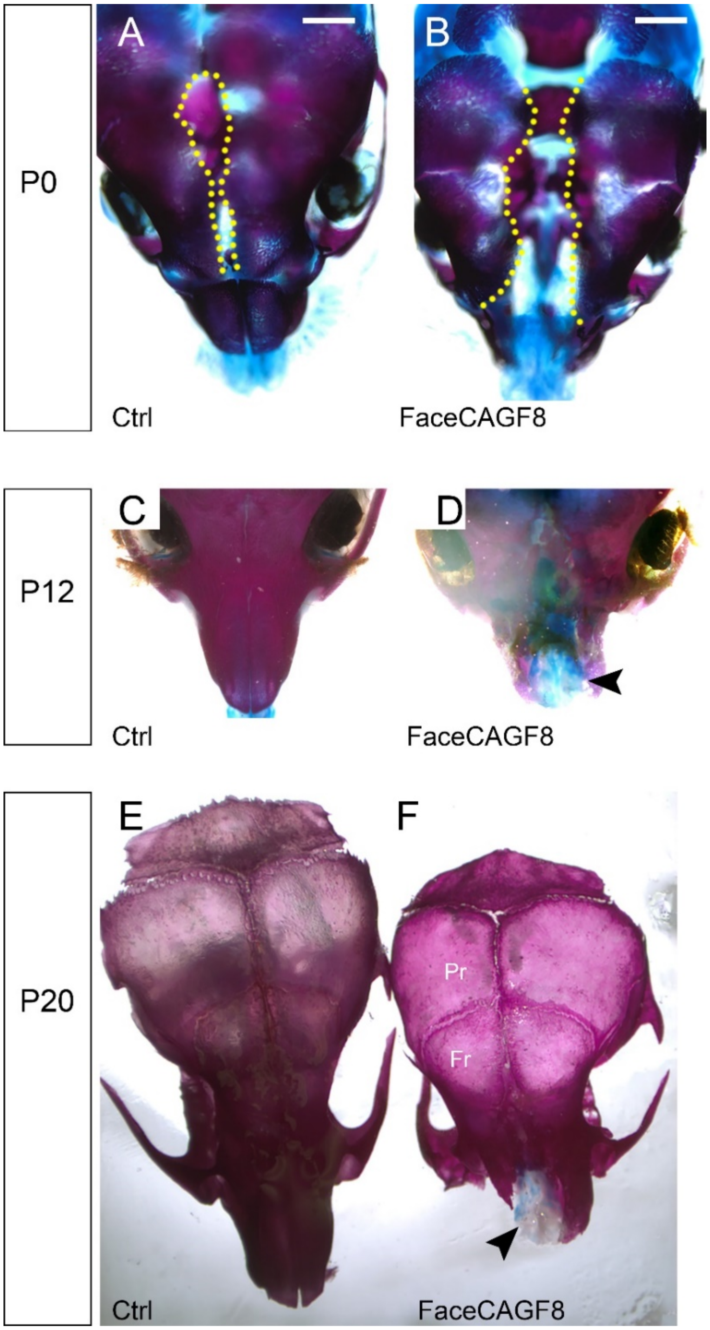

Supplemental Figure 13. ***FaceCAGF8*** **mice skeletal defects.**

Bone and cartilage staining of control (A, C, E) and *FaceCAGF8* (B, D, F) skulls at P0 (A-B), P12 (C-D), and P20 (E-F), dorsal view. Yellow dotted lines note the boundary of the bone. Black arrowheads denote abnormal tissue in the region that should be occupied by the nasal bones. Abbreviations: Fr, frontal; Pr, parietal. Scale Bars= 1 mm.

Supplemental Tables

Supplemental Table 1. Gene comparison of Wildtype Cranial Vault vs. *MCAGF8* Unless otherwise specified, all data are derived from comparing wildtype (WT, Control) to *MCAGF8* (Mutant, Treatment) cranial vault. Cranial vault tissue was collected from 9 E14.5 control and mutant embryos as outlined in Fig. 7.

Tab1: All genes reported from RNA-seq sorted by ENSEMBL ID. Means are expressed as RPKM.

Tab2: Significant genes ($p<0.05$), sorted by fold change. Blue highlighted cells are genes downregulated <-1.5; yellow highlighted cells are genes upregulated >1.5.

Tab3: Genes listed in text, sorted by order of appearance. Left: wildtype cranial vault vs mutant cranial vault; Right: wildtype cranial base vs mutant. Blue highlighted cells are genes downregulated <-1.5; yellow highlighted cells are genes upregulated >1.5. Significant P-values ($p<0.05$) highlighted in green.

Tab4: Significant genes ($p<0.05$), sorted by control mean, with a control mean RPKM >100 and have an expression fold change of either >1.35 (upregulated) or <-1.35 (downregulated). Blue highlighted cells are genes downregulated <-1.5; yellow highlighted cells are genes upregulated >1.5.

Tab5: Significant genes ($p<0.05$), sorted by treatment (mutant) mean, with an *MCAGF8* mean RPKM >100 and an expression fold change of either >1.35 (upregulated) or <-1.35 (downregulated). Blue highlighted cells are genes downregulated <-1.5; yellow highlighted cells are genes upregulated >1.5.

Tab6: Relevant values and histogram plot of normalized RPKM values of genes associated with BMP signaling and significantly dysregulated in *MCAGF8* cranial vault samples (orange) as compared to controls (blue). Error bars represent relative standard error calculated from 3 replicates.

Tab7: Relevant values and histogram plot of normalized RPKM values of genes associated with Hedgehog signaling and significantly dysregulated in *MCAGF8* cranial vault samples (orange) as compared to controls (blue). Error bars represent relative standard error calculated from 3 replicates.

Tab8: Top functional annotation clusters as calculated by DAVID using all significant genes ($p<0.05$).

Tab9: Top functional annotation charting as calculated by DAVID using significant genes ($p<0.05$) that have a fold change of >1.5 or <-1.5.

Click here to Download Table S1

Supplemental Table 2. Gene comparison of Wildtype Cranial Base vs. *MCAGF8* cranial vault

Unless otherwise specified, all data are derived from comparing wildtype cranial base (WT, Control) and *MCAGF8* (Mutant, Treatment) cranial vault tissue. Cranial tissue was collected from 9 E14.5 control and *MCAGF8* embryos as outlined in Fig. 7.

Tab1: All genes reported from RNA-seq sorted by ENSEMBL ID. Means are expressed as RPKM.

Tab2: Significant genes ($p<0.05$), sorted by fold change. Blue highlighted cells are genes downregulated <-1.5; yellow highlighted cells are genes upregulated >1.5.

Tab3: Significant genes ($p<0.05$), sorted by control mean, with a control mean RPKM >100 and have an expression fold change of either >1.35 (upregulated) or <-1.35 (downregulated). Blue highlighted cells are genes downregulated <-1.5; yellow highlighted cells are genes upregulated >1.5.

Tab4: Significant genes ($p<0.05$), sorted by treatment (mutant) mean with a *MCAGF8* mean RPKM >100 and an expression fold change of either >1.35 (upregulated) or <-1.35 (downregulated). Blue highlighted cells are genes downregulated <-1.5; yellow highlighted cells are genes upregulated >1.5.

Tab5: Top functional annotation clusters as calculated by DAVID using all significant genes ($p<0.05$).

Tab6: Top functional annotation charting as calculated by DAVID using significant genes ($p<0.05$) that have a fold change of >1.5 or <-1.5.

Click here to Download Table S2

Disease Models & Mechanisms • Supplementary information

Primer Name	Purpose	Sequence
Fgf8b FWD	Cloning	GGATC CCTCG AGCGC GCCAT GGGCA GCCCC CGCTC C
Fgf8b REV	Cloning	CGAGC TGAAG CTTCG CCTAT CGGGG CTCCG GGGCC CAAG
BTR	ES Screening/ Genotyping	CTAGA GCGGC CTCGA CTCTA CGATA CCGTC GATCC CC
R5F	ES screening, outside 5' homology	GGCTG TGCTT TGGGG CTCCG GCTCC TCAG
GFP F	ES screening	CCAAC GAGAA GCGCG ATCAC ATGGT CCTGC TGGAG TTCGT G
R3R	ES screening, outside 3' homology	CCTCA GAGAA ATGGA GTAGT TACTC CACTT TCAAG TTCC
CMV R1	ES screening/ Genotyping	CGTTG GGCGG TCAGC CAGGC GGGCC ATTTA CCG
Rosa F	Genotyping	GGGAG TTCTC TGCTG CCTCC TGGCT TCTGA GG
Rosa R	Genotyping	CCTGC AGGAC AACGC CCACA CACCA GG
Cre1	Cre Genotyping	GCTGG TTAGC ACCGC AGGTG TAGAG
Cre3	Cre Genotyping	CGCCA TCTTC CAGCA GGCGC ACC
oIMR0039	LacZ Genotyping	ATCCT CTGCA TGGTC AGGTC
oIMR0040	LacZ Genotyping	CGTGG CCTGA TTCAT TCC
Fgf8-F	qRT-PCR	CCGGA CCTAC CAGCT CTACA
Fgf8-R	qRT-PCR	GGCAA TTAGC TTCCC CTTCT
Bactin-qF	qRT-PCR	GCGAG CACAG CTTCT TTG
Bactin-qR	qRT-PCR	CCATG TTCAA TGGGG TACTT C

Supplemental Table 3. Primer Names and Sequences

Primer name, purpose, and sequences for all primers utilized.

Disease Models & Mechanisms • Supplementary information
